# When do we care about political neutrality? The hypocritical nature of reaction to political bias

**DOI:** 10.1371/journal.pone.0196674

**Published:** 2018-05-03

**Authors:** Omer Yair, Raanan Sulitzeanu-Kenan

**Affiliations:** 1 Department of Political Science, Stony Brook University, Stony Brook, NY, United States of America; 2 Department of Political Science and the Federmann School of Public Policy and Government, The Hebrew University of Jerusalem, Jerusalem, Israel; Rice University, UNITED STATES

## Abstract

Claims and accusations of political bias are common in many countries. The essence of such claims is a denunciation of alleged violations of political neutrality in the context of media coverage, legal and bureaucratic decisions, academic teaching etc. Yet the acts and messages that give rise to such claims are also embedded within a context of intergroup competition. Thus, in evaluating the seriousness of, and the need for taking a corrective action in reaction to a purported politically biased act people may consider both the alleged normative violation and the political implications of the act/message for the evaluator’s ingroup. The question thus arises whether partisans react similarly to *ingroup-aiding* and *ingroup-harming* actions or messages which they perceive as politically biased. In three separate studies, conducted in two countries, we show that political considerations strongly affect partisans’ reactions to actions and messages that they perceive as politically biased. Namely, ingroup-harming biased messages/acts are considered more serious and are more likely to warrant corrective action in comparison to ingroup-aiding biased messages/acts. We conclude by discussing the implications of these findings for the implementations of measures intended for correcting and preventing biases, and for the nature of conflict and competition between rival political groups.

## Introduction

The prevalence of accusations and condemnations of political bias in many countries (see, e.g., [1]; see more below), reflect a widespread social reality in which actions and messages that are perceived as politically biased, are considered inappropriate and even morally reprehensible. People tend to understand the term "political bias" as "lack of neutrality" [2] and as unfair favoritism towards one political group, candidate or ideology [3]; and the pejorative connotation of "political bias"–much like that of "gender bias" or "racial bias"–suggests that whenever possible, politically biased acts should be identified as such, and a corrective action should be taken. Indeed, the essence of claims of political bias is a denunciation of apparent violations of political neutrality in settings where this norm is expected to apply–e.g., media coverage [4,5], legal and bureaucratic decisions [6–8], or academic teaching [9,10].

Yet, as we explain in full below, politically biased acts elicit not only normative considerations but also *political* considerations. Political biases are embedded within a context of intergroup competition, thus a biased action or message can pose a threat to a particular political group or ideology, and concurrently benefit its rival group(s) or advance a competing ideology. Given the normative nature of political bias, one may expect partisans to react similarly to ingroup-aiding and ingroup-harming biases–as both appear to violate the neutrality norm. Conversely, partisans whose group stands to gain or lose from a certain politically biased act, may be politically motivated to react differently to ingroup-aiding and ingroup-harming biases. Thus, a question arises, to what extent normative considerations affect people’s concerns about political bias as oppose to their political motivations.

In this article, we set to empirically address this question by comparing the reactions of partisans to either ingroup-aiding and ingroup-harming biased messages. Drawing on the motivated reasoning literature in general [11] and on its consequences for normative and moral judgment in particular [12], we hypothesize that partisans who perceive an ingroup-harming biased message will consider the bias as more serious and will be more likely to seek corrective action, in comparison to partisans who perceive an ingroup-aiding biased message. In three studies–two conducted in Israel, that tapped reactions to perceived media bias, and another study, conducted in the U.S., that recorded reactions to allegations of political bias leveled against the social network Facebook–we provide support for this hypothesis. Our results have important implications for the nature of conflicts between rival political groups. These results cast doubt on the likelihood of inter-partisan cooperation in addressing political bias, even in cases where both sides agree about the existence of bias. Moreover, such divergent reactions to politically biased acts could even further aggravate intergroup relations.

## Political bias and inter-group rivalry

People overwhelmingly express disapproval of political bias in various social contexts, and many consider political bias to be a problem. People overwhelmingly report preferring neutrality in various social institutions over political bias, including a bias *in favor* of their own group. For example, in the U.S. a clear majority among Republicans, Independents, and Democrats prefer that congressional districts will be drawn with "no partisan bias whatsoever" even if such bias would help their preferred party win more seats [13]. Similarly, a clear majority among Republicans, Independents, and Democrats report that they prefer to get news from sources that are neutral and have no particular political point of view, than from sources that share their political views [14].

Moreover, "political bias" in various institutions is considered a grave problem. For example, when asked about problems facing American higher education, roughly 40 percent of Americans considered political bias in the classrooms as "a very serious problem" (p. 11 in [7]). In a recent survey in Israel a majority of the respondents considered political bias as the Israeli media's biggest problem (p. 107 in [15]). Relatedly, several studies suggest that perceptions of political bias in the media, in particular bias against one's ingroup, have detrimental ramifications, including reduced trust in the media and in democratic institutions, and a feeling of alienation from society (see, e.g., [2,16,17]).

Accordingly, it should come as no surprise that allegations of bias often involve an implicit or explicit demand for a corrective act, or, at the very least, for reverting to fair and evenhanded conduct henceforth. For example, people who identity a certain news item as biased against their ingroup take various "corrective" actions–such as sending e-mails and posting online comments concerning the news item–in order to try and counteract the bias [18,19]. Moreover, several scholars have noted that "concerns about bias have been a central driver of media regulation" (p. 2 in [20]; see also p. 44 in [21]). Relatedly, those who accused in May 2016 the social media company Facebook of political bias (e.g., [22]) demanded that Facebook would eliminate that bias (e.g., [23]; see more below), and people who believe that political bias in academia constitutes a problem also think it should be addressed (p. 11 in [9]; relatedly, see [24]).

People's reactions to political bias thus carry important social and political implications. Moss-Racusin and colleagues have noted that "in many ways, evidence of bias is only as impactful as the responses it engenders" (p. 206 in [25]), as such reactions constitute the first step to correcting the situation and for working to prevent future biased acts. However, while "political bias" denotes a violation of a social norm, acts and messages that elicit claims of political bias occur within the context of intergroup rivalry. A biased action or message that harms a particular political group or ideology, concurrently benefit its rival group(s) or advance a competing ideology. Still, when asked about these issues in surveys, people generally state that they prefer neutrality to bias even if that bias would assist their party or ideology (see above). In this research we provide evidence on the extent to which political motivations affect people’s actual reactions (rather than reported preferences) to political bias.

An important clarification is in order at this point. Some studies have shown that rival partisans often disagree on the existence and/or direction of political bias, i.e., which group is unfairly (dis)favored by a certain action or message [17,26]. However, these differences in perceptions are not our concern here. Indeed, we note that many studies have investigated the antecedents of people's perceptions of political bias–mostly in news coverage–since the 1980s (for recent reviews, see [17,27]). Instead, we are interested in the *reactions* of rival partisans to political bias when both sides similarly identify the message or act in question as biased against (or in favor of) of a certain group. Given that the essence of claims of political bias is a denunciation of violations of political neutrality, we would expect that concern for political neutrality–reflected by perceiving an act or message as politically biased–would elicit similar reactions across the political divide. On the other hand, despite the normative basis of accusations of political bias, rival partisans might act in a hypocritical manner, and would adjust their reactions to politically biased acts based on inter-group rivalry considerations. A theoretical basis for the latter behavior lay in motivated reasoning theory.

### Motivated reasoning in shaping reactions to political bias

The motivated reasoning literature suggests that people are affected by their motivations and desires when they process information and make judgments [11,28,29]. In particular, various studies have shown that people's motivations affect *normative and moral* judgments [12,30]. The literature on "moral hypocrisy" has shown that one's own moral transgressions are seen as less immoral than identical transgressions committed by another person [31]. Furthermore, people tend to evaluate immoral behavior committed by others more harshly when they do not stand to gain from it, in comparison to when they stand to gain from it [32].

Pertaining more specifically to political rivalries, scholars have noted that people "tend to evaluate information and make judgments in a manner that best serves the interests of groups to which they belong" (p. 1110 in [33]). Such group-based evaluations are shaped by a partisan's motivation to protect the ingroup and help it in the context of competitive interactions. For example, partisans evaluate unethical behaviors committed by their fellow partisans or by politicians from their party as less serious and more justified than identical behaviors committed by members of the rival group [34–36].

As previous studies have shown, the effects of partisans' motivations on their judgments and behaviors is rooted in partisans' affective attachments to their ingroup [37–39]. Thus, in the context of this study we can expect that identifying an ingroup-aiding politically biased act would not cause partisans to experience a negative emotional reaction as they would experience when identifying an ingroup-harming politically biased act. These potential differences in emotional reaction may engender different evaluations of both the seriousness of the biased act, and the extent to which it requires a correcting intervention.

We therefore expect that, despite the normative basis of accusations political bias, partisans would react differently to ingroup-aiding and ingroup-harming biased acts. Namely, partisans who identify a biased act that favors their ingroup would consider it as less severe and less warranting corrective action compared to an ingroup-harming biased act. The ingroup-aiding biased act, while presumably amiss and reprehensible, would fail to elicit the same emotional reaction as an ingroup-harming biased act. Stated formally, we hypothesize that:

*Hypothesis 1*: *Partisans who identify an ingroup-aiding political bias will be less likely to take corrective action and will consider it less serious than partisans who identify an ingroup-harming political bias*.

Before moving to our empirical analyses, we should make it clear that since we lack an objective yardstick to gauge the true level of seriousness and need for correction of whichever political bias, we do not claim to determine whether partisans who identify ingroup-aiding bias or partisans who identify ingroup-harming bias are *correct* in their evaluations and reactions. Rather, we focus on the discrepancies between partisans' reactions to these two ingroup-based "types" of bias. Accordingly, in all three studies, we compare the results of rival partisans. Moreover, because we focus in this paper on partisans' reactions to bias, we do not elaborate in the main text about non-partisans' reactions to bias, and in various sections in S1 Appendix we compare the evaluations of partisans and non-partisans.

## Empirical analyses

### Overview of the present studies

Three studies, involving 886 participants, were conducted to test our hypothesis (all studies in this paper have been approved by the Ethics Committee of the Faculty of Social Sciences at the Hebrew University of Jerusalem). Study 1, a survey among Israeli citizens, served as a preliminary test of the hypothesis. In Study 2 we aimed to replicate the results in a different country and context, while using an additional dependent variable. Finally, to test the causal relationship envisaged by our theory, Study 3 experimentally treated the partisan orientation of the messages presented to respondents, thereby randomly assigning them to experience either a biased message in favor of their ingroup or against their ingroup. The results of the three studies provide externally and internally valid strong support for our hypothesis.

### Study 1

#### Overview

In this study respondents read a news article and answered several questions about it. The survey was fielded between January 27 and February 18, 2016.

#### Participants

A total of 346 respondents, recruited from *Panel Hamidgam*, a survey company conducting online surveys in Israel, participated in the survey (response rates for the three studies are reported in Section A in S1 Appendix). Our sample is not a representative sample of the Israeli population, yet it is quite diverse. Average age was 38.8 (*SD* = 12.6) and women constituted 46.8% of the sample. A plurality of respondents (41.3%) reported voting in the recent Israeli national elections (March 2015) for right-wing parties, 24.8% for left-wing parties, 22.6% for center parties, and 11.3% either reported voting for other parties, not voting, or refused to answer (for more details on the sample, see Section A in S1 Appendix).

#### Procedure

Respondents were told they were going to participate in a short study in which they would evaluate a news article. They read a 350-word article, presented as a news article published during October 2015 in one of Israel's top online news websites. The article elaborated about the actions and statements of Israel's Prime Minister, Benjamin Netanyahu, following a series of violent attacks by Palestinians against Israelis, which had occurred several days earlier (Section B in S1 Appendix includes the texts of all the articles used in the three studies). After reading the article, respondents answered a question regarding bias in the article, and a follow-up question regarding the need to correct the article (details in the following paragraph). Finally, respondents answered several questions addressing demographics and political attitudes.

#### Measurements

In order to tap perceptions of bias in the article, we asked respondents a question commonly used in previous studies (e.g., [40–42]) and adapted to the specific context–whether the article was neutral, or whether it was biased in favor or against Netanyahu. This item was followed by a 7-point scale anchored by 1, *Biased in favor of Netanyahu*, 4, *Neutral article*, and 7, *Biased against Netanyahu*. Some studies have used two or more items in tapping perceptions of bias in an article (e.g., [40]). We chose not to do so for methodological reasons, as the next question, tapping our main dependent variable, could have been asked as a follow-up to only one question. That said, typical responses to a question about bias in a news article have been shown to be associated with normative evaluations such as whether the article is fair, whether it can be trusted, and whether is it factual or false (see, e.g., [43–45]). The article was generally identified as slightly biased against Benjamin Netanyahu (*M* = 4.38; *SD* = 1.46), with right-wing voters identifying more bias against Netanyahu (*M* = 4.68; *SD* = 1.34) than left-wing voters (*M* = 4.14; *SD* = 1.48) (*t*(151.27) = 2.65; *p* = .009).

While this question taps bias in favor of, or against Netanyahu, in order to test our hypothesis we needed a measure of the perceived "type" of bias, that is, whether partisans identify the bias–in case they identify any bias in the article–as ingroup-aiding or as ingroup-harming. We created such a measure by relating respondents' answers to the abovementioned 7-point "bias in the article" question with their vote choice in the previous election. Israel is a multi-party system, but it currently has several recognized political blocs, namely, the right-wing, center, and left-wing blocs [46]. And since the main policy dimension in Israeli politics is the hawk vs. dove approach to the Israeli-Arab conflict (e.g., [47]), in this study we consider voters of right- and left-wing parties as partisans, and voters of center parties as non-partisans. Notably, at the time of the survey, Netanyahu headed the country’s biggest party, the right-wing Likud party, as well as a right-wing coalition government.

Accordingly, the combinations of partisans' vote choice and their perceptions regarding the article–as either neutral (not biased) or biased against/in favor of Netanyahu–were used to engender three categories for a *Bias Type* measure, which constitutes our main independent variable: ingroup-aiding bias; no bias [neutral]; and ingroup-harming bias. For example, voters of right-wing (left-wing) parties who perceived the article as biased to any degree in favor of (against) Netanyahu were categorized as identifying an *ingroup-aiding bias*. In contrast, voters of right-wing (left-wing) parties who perceived bias to any degree against (in favor of) Netanyahu were categorized as identifying an *ingroup-harming bias*. Overall, 16.4% of partisans identified ingroup-aiding bias, 47.6% identified no bias, and 36.1% identified ingroup-harming bias.

Following the "bias in the article" question, respondents answered a novel question that constitutes our dependent variable–*Demand for Correction*. Respondents were first reminded of the answer they had just given in the "bias in the article" question (neutral or biased against/in-favor of Netanyahu), and then were asked: "Considering your previous answer, if you were the editor in charge of publishing this article, would you approve the article as is, or would you demand to correct it prior to publication?" (Response options: approve, demand correction, don't know) (see Study 1 in [48] for a somewhat similar measure). This question was intended to provide a measure of people's reaction to bias, specifically the degree to which they saw a need for corrective action. Overall, 23.5% of partisans demanded correction. In the statistical analyses reported in the main text (i.e., from all three studies) we consider respondents who answered "don't know" to the "correction" question as not demanding a corrective action. As shown in various sections in S1 Appendix, omitting these respondents from either study hardly affects the results.

#### Results and discussion

We begin with the raw proportion of partisans demanding correction of the article in each of the three *Bias Type* categories. These results provide initial support for our hypothesis: among partisans (i.e., voters of left/right-wing parties) who identified a neutral article, 6.1% [95% CIs: 1.3%, 10.8%] demanded a correction; among partisans who identified an ingroup-aiding bias, this proportion rises to 20.6% [6.8%, 34.4%], and among partisans who identified an ingroup-harming bias, this proportion increases further to 49.3% [37.9%, 60.7%]. The difference between the three groups is statistically significant (*χ*^2^(2) = 44.02; *p* < .001, two-tailed tests throughout). We also fitted a logistic regression with *Demand for Correction* regressed on dummy variables for the ‘ingroup-aiding bias’ and ‘ingroup-harming bias’ groups (‘no bias’ as reference category). Results are presented in Model 1 of Table 1. The coefficients of both dummy variables are significantly different from the ‘no bias’ reference category. Notably, the difference between the ‘ingroup-aiding bias’ and ‘ingroup-harming bias’ groups is statistically significant (*p* = .006), with partisans reporting ingroup-harming bias more likely to demand a corrective action than partisans reporting ingroup-aiding bias.

**Table 1:** Study 1 –Determinants of Demand for Correction.

	(1)	(2)	(3)
Dependent Variable	Demand for Correction	Demand for Correction	Demand for Correction
Ingroup-aiding Bias	1.391*	0.854	0.926
	(0.599)	(0.711)	(0.749)
Ingroup-harming Bias	2.714***	2.217***	2.217***
	(0.482)	(0.551)	(0.576)
Moderate Bias		0.720	0.804
		(0.498)	(0.526)
Strong Bias		0.933+	1.162+
		(0.520)	(0.600)
Constant	-2.741***	-2.741***	-2.466
	(0.422)	(0.422)	(1.749)
Coefficient equality F-tests (p-value)			
H0: Ingroup-aiding Bias = Ingroup-harming Bias	.006	.007	.026
Individual-level Cov.	NO	NO	YES
Observations	208	208	204
Pseudo-R^2^	0.199	0.216	0.260

*Note*. Robust standard errors in parentheses

*** p<0.001

** p<0.01

* p<0.05

+ p<0.1.

The *Ingroup-aiding Bias* and the *Ingroup-harming Bias* dummy variables are based on the reported vote in Israeli 2015 national election, together with the 7-point "bias in the article" question.

A potential confounder is the amount, or level of bias each respondent identified in the article. For example, those reporting a strong bias in favor of/against Netanyahu (e.g., 1 on the 7-point "bias in the article" scale) are arguably more likely to demand a correction than those reporting weak bias in favor of/against Netanyahu (e.g., 3 on that scale). This is true regardless of whether bias is perceived as ingroup-aiding or ingroup-harming. Thus, we created a *Level of Bias* variable by folding the 7-point "bias in the article" scale at the midpoint (4). The mean score of this variable among partisans was 0.99 (*SD* = 1.12) on a 0–3 scale. It should be noted that the ‘no bias’ category is also the lowest score (0) in the *Level of Bias* variable, and accordingly we need to differentiate the effect of the type of the bias (either ingroup-aiding or ingroup-harming) from the effect of the *Level of Bias* variable. To do so, we created two dummy variables denoting *Moderate Bias* (2 or 6 on the 7-point "bias in the article" scale) and *Strong Bias* (1 or 7 on that scale), with the reference category being ‘no bias’ or a ‘weak bias’ (3, 4, or 5 on that scale). By including these two dummy variables our estimate for the effect of our main independent variable, *Bias Type*, is above and beyond the effect of perceived level of bias.

**Fig 1:**
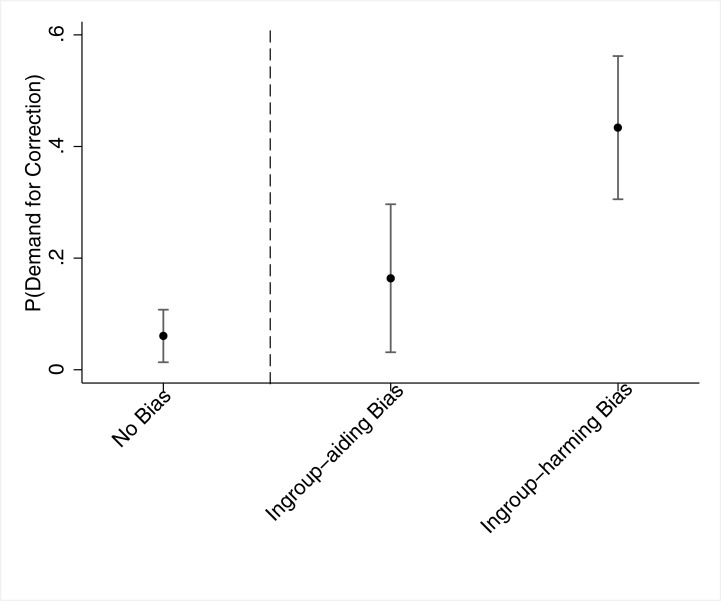
Study 1 –Predicted probabilities of Demand for Correction across partisans, by the different "types" of bias. *Note*. The dots denote point estimates, and the whiskers denote 95% confidence intervals. The dashed vertical line separates between those who did not identify any bias in the article and those who identified it as biased (either against or in favor of their ingroup).

**Fig 2:**
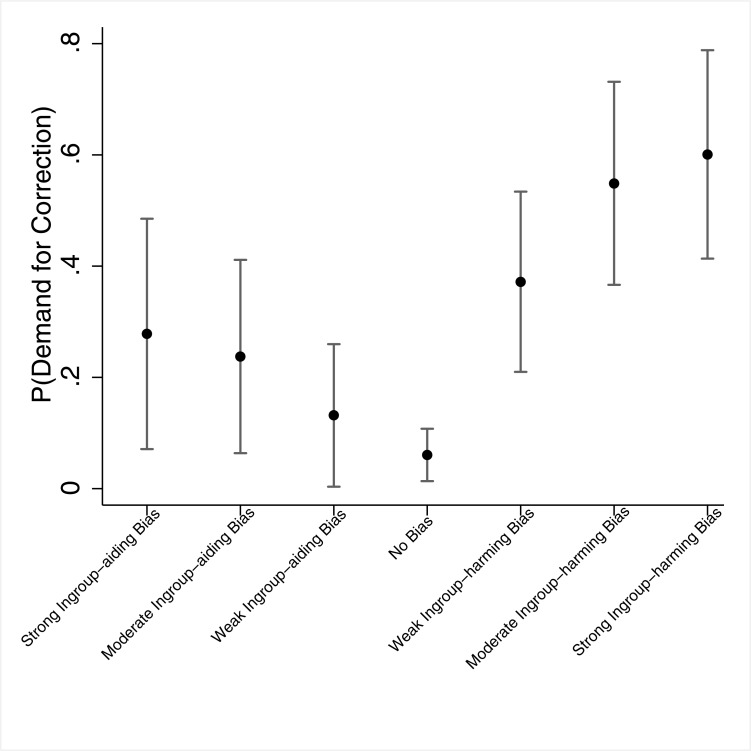
Study 1 –Predicted probabilities of Demand for Correction across partisans, by both the "type" and level of bias. *Note*. The dots denote point estimates, and the whiskers denote 95% confidence intervals.

Model 2 includes these two dummy variables. The coefficient of the ‘ingroup-aiding bias’ dummy variable is no longer statistically significant (*p* = .229), while the coefficient of the ‘ingroup-harming bias’ dummy variable remains statistically significant, with the difference between the coefficients of these two variables statistically significant (*p* = .007). Substantively, when controlling for the level of bias reported by the respondent, we see that it is mostly identifying an *ingroup-harming* bias that motivated partisans to correct a biased news article, rather than identifying bias *per se*. The predicted probabilities of demanding a correction based on Model 2 are graphically shown in Figs 1 and 2. Fig 1 shows that 16.4% [3.1%, 29.7%] of partisans who identified bias in favor of their political camp demanded a corrective action (holding *Level of Bias* at its mean), in comparison to 6.1% [1.3%, 10.8%] of partisans who identified no bias at all. Among partisans who identified bias against their political group, however, the probability of demanding a correction (holding *Level of Bias* at its mean) was 43.4% [30.6%, 56.2%].

In Fig 2 we calculated the predicted probabilities of demanding a correction for each of the seven possible levels of perceived bias–from a strong ingroup-aiding bias to a strong ingroup-harming bias. As clearly shown, identifying any level of ingroup-harming bias invoked a demand for correction of the article at a higher rate than identifying no bias or any level of ingroup-aiding bias. In addition, in all three pair-wise comparisons between partisans who identified ‘ingroup-aiding bias’ and ‘ingroup-harming bias’ at the same level of bias (e.g., weak ingroup-aiding bias compared to weak ingroup-harming bias), those in the ‘ingroup-harming bias’ group demanded a correction at higher rates than those in the ‘ingroup-aiding bias’ group (*p*s < .015), providing further support for our hypothesis.

In Model 3 we added various individual-level variables (e.g., age, gender, political interest). This hardly affected our results (the full results are detailed in Section C in S1 Appendix), suggesting their robustness not only to selection on observables but also to selection on unobservables as proposed in [49]. In addition, in a series of robustness tests, detailed in Section C in S1 Appendix, we show that alternative model specifications do not substantially alter our main results. These results provide initial support for our hypothesis that partisans, identifying political bias that is harmful to their political group, are more likely to demand corrective action than partisans identifying an ingroup-aiding bias. Still, there are clear limitations to this study. First, it was conducted in one country and in one (media) context. Second, respondents self-selected into either ingroup-aiding or ingroup-harming bias condition. These limitations are addressed by Studies 2 and 3, respectively.

### Study 2

#### Overview

In May 9, 2016, the internet website Gizmodo published an article in which a former worker of the popular social network Facebook claimed that Facebook workers routinely suppressed news items that would be of interest to conservative users in the company's "trending" news section [22]. The report quickly received media attention, with conservatives accusing Facebook of political bias and calling on it to investigate the allegations (e.g., [23]). Facebook officials were quick to deny the allegations, and within two weeks the company reported that it had conducted an investigation in which no evidence of bias was found, but that the company would nonetheless change the news feature so as to reduce human judgment in the feature's operation [50]. We took advantage of this event to investigate whether conservatives and liberals in the U.S. exhibit similar patterns of reactions to allegations of political bias. The survey was fielded between May 23 and 29, 2016.

#### Participants

One hundred and ninety-six U.S. respondents, recruited from Amazon's Mechanical Turk (MTurk), participated in the survey. MTurk is considered a valid recruitment tool for research on political ideology [51], and we recruited MTurk workers with approval rates of more than 95% in previous tasks, and more than 500 previous MTurk tasks, as such workers are known to provide high data quality [52]. Our sample is not a representative sample of U.S. population, yet it is relatively diverse, and its characteristics resemble those of other recent MTurk samples (see Section A in S1 Appendix). Average age was 37.3 (*SD* = 10.8) and women constituted 43.4% of the sample. A majority of respondents (54.6%) identified as ideologically liberal, 26.0% as conservatives, and 18.9% as moderates (one respondent [0.5%] answered "don't know" to this ideology question).

#### Procedure

The task was advertised on MTurk as "a short public opinion survey", and the survey's opening statement indicated that it was a short survey concerning a certain news item. Participants answered several demographic and political questions, and were then presented with a brief, 200-word summary, which described both the allegations against Facebook and the company’s response. We then asked respondents several questions regarding this issue, including three questions concerning their attitudes towards these allegations if they were found to be true.

#### Measurements

In this study, unlike study 1, respondents were asked to evaluate *allegations* of political bias (rather than to evaluate bias in a news article). Accordingly, we first asked respondents about their belief in the truthfulness of these allegations. Respondents were asked, "To what degree do you believe that these allegations of discrimination against conservative topics in Facebook's news section are true?" That item was followed by a 5-point scale anchored by 1, *To no degree*, 3, *To* s*ome degree*, and 5, *To a very large degree*. Overall, our sample believed these allegations to some degree (*M* = 2.98; *SD* = 1.21), but, perhaps not surprisingly, liberal and conservative respondents differed in their level of belief: liberals believed these allegations to a lesser degree than conservatives (*M*_*Liberals*_ = 2.58, *SD* = 1.08; *M*_*Conservatives*_ = 3.61; *SD* = 1.10) (*t*(98.04) = -5.37; *p* < .001).

These different levels of belief in the allegations are telling, yet they do not help us tap people's reactions to political bias in the case they identified one. Accordingly, we asked respondents to answer the following questions under the assumption that these allegations were true. In the next question respondents were asked, "If it is discovered that these allegations are true, would you consider these actions of Facebook's workers as political bias against conservatives?" (Response options: yes, no, don't know). Due to the specific circumstances in which Facebook was accused of bias only against conservatives, we did not consider it reasonable to include in this question an option of bias against liberals.

Next, we asked respondents two questions (presented in random order) that constitute our dependent variables. One question (*Change Algorithm*) asked, "If it is discovered that these allegations are true, do you think that Facebook should change the way it selects news items appearing in its ‘‘trending” news section?" (Response options: yes, no, don’t know). This question was intended to be similar to Study 1's dependent variable. Overall, 69.4% of respondents thought Facebook should change its algorithm if the allegations were true.

The second question (*Seriousness*) asked, "If it is discovered that these allegations are true, how serious would you say these actions of Facebook's workers are?" That item was followed by a 7-point scale anchored by 1, *Not at all serious*, 4, *Somewhat serious*, and 7, *Very serious* (see also [34,36]). Overall mean response described the actions as somewhat serious (*M* = 4.03; *SD* = 1.78). As we expected, the *Seriousness* and *Change Algorithm* questions were positively correlated (*Spearman rho* = .50; *p* < .001). It should be noted that the *Seriousness* variable is not the same as the *Level of Bias* variable used in Study 1. The *Seriousness* variable captures an assessment of the *normative severity* of Facebook workers’ actions, whereas the *Level of Bias* variable captures the *extent* of bias against (in favor of) a particular person or group.

In creating our independent variable, *Bias Type*, we used the standard 7-point liberal-conservative ideological scale (1- extremely liberal, 7- extremely conservative), taken from the American National Election Study (see http://www.electionstudies.org/). Again, the *Bias Type* variable had three categories: *ingroup-aiding bias*, *no bias*, and *ingroup-harming bias*. Since Facebook was accused of bias against conservatives, liberals (1–3 on the ideology scale) who considered the actions as bias against conservatives (if allegations were true) were assumed to experience an *ingroup-aiding bias*, while conservatives (5–7 on the ideology scale) who considered the actions as bias against conservatives were assumed to experience an *ingroup-harming bias*. Both liberals and conservatives who did not considered the actions as biased against conservatives were assumed to experience *no bias*.

#### Results and discussion

Overall, 74.5% of respondents considered Facebook’s alleged actions as political bias against conservatives (if allegations were true). A significant difference between liberals and conservatives emerged, with 92.2% [84.7%, 99.6%] of conservatives considering these actions political bias, in comparison to only 65.4% [56.4%, 74.5%] of liberals (*χ*^2^(1) = 12.85; *p* < .001). This difference could attest to ideological bias on behalf of either liberals and/or conservatives; yet, importantly, even among liberals there was a clear majority who characterized these alleged actions as political bias against conservatives. This corroborates the suggestion that a clear bias against (or in favor of) a certain group would be seen as such by all groups (see also [42,53]).

**Fig 3:**
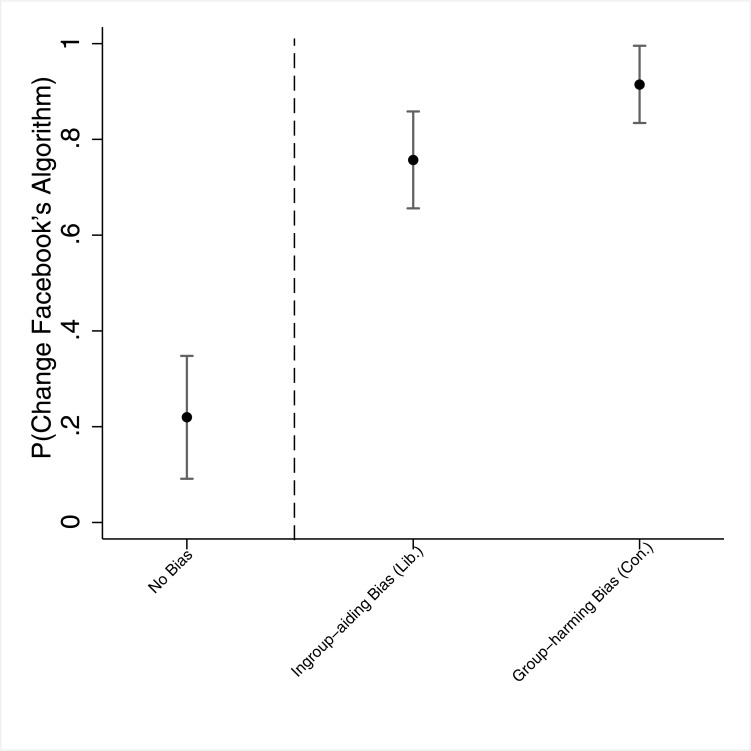
Study 2 –Changing Facebook's algorithm, by the different "types" of bias. *Note*. The dots denote point estimates, and the whiskers denote 95% confidence intervals. "Lib." denotes liberals, and "Con." denotes conservatives. The dashed vertical line separates between those who did not consider the Facebook's actions as bias and those who consider it as biased (either ingroup-aiding bias [liberals] or ingroup-harming bias [conservatives]).

Turning to our first dependent variable, *Change Algorithm*, we present our results separately for the three *Bias Type* categories. Among those in the *no bias* group, only 22.0% [9.1%, 34.8%] thought that Facebook should change its algorithm if the allegations are found true. A much higher proportion in the *ingroup-aiding bias* and *ingroup-harming bias* groups thought Facebook should change its algorithm, yet while among those in the *ingroup-aiding bias* group (liberals who considered Facebook workers' actions as bias against conservatives) 75.7% [65.6%, 85.8%] thought a change to Facebook's algorithm is in place, in the *ingroup-harming bias* group (conservatives who considered Facebook workers' actions as bias against conservatives) that figure was 91.5% [83.4%, 99.6%]. The difference between the three groups is statistically significant (*χ*^2^(2) = 52.33; *p* < .001), and, importantly, the difference between the *ingroup-aiding* and *ingroup-harming* groups is also statistically significant (*χ*^2^(1) = 4.75; *p* = .029). These results are graphically shown in Fig 3. In Section E in S1 Appendix we provide the results of several regression analyses and robustness tests which show that the results are not sensitive to a range of model specifications. In Section E in S1 Appendix we also show that the substantive results for both dependent variables hold when comparing across republicans and democrats, instead of conservatives and liberals.

**Fig 4:**
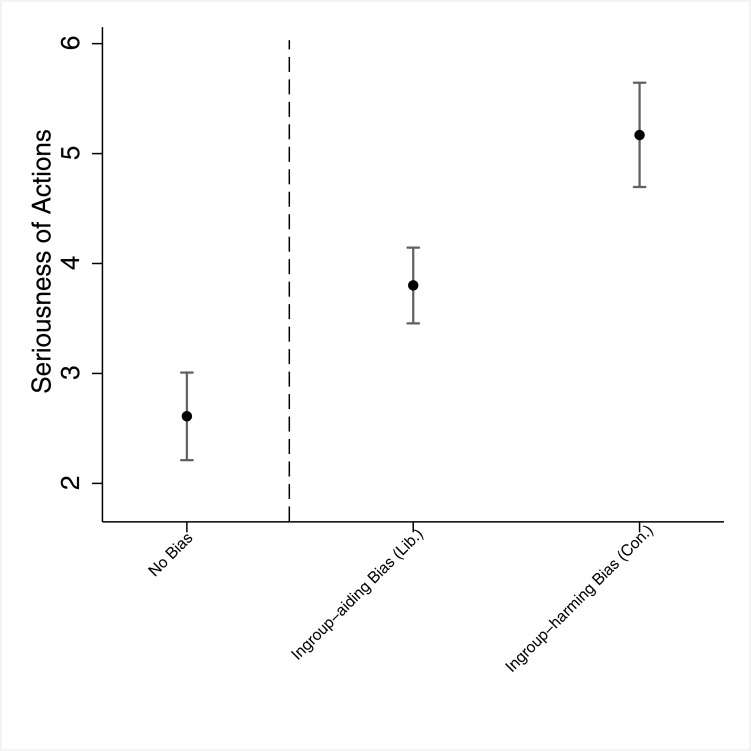
Study 2 –Seriousness ratings, by the different "types" of bias. *Note*. The dots denote point estimates, and the whiskers denote 95% confidence intervals. "Lib." denotes liberals, and "Con." denotes conservatives. The dashed vertical line separates between those who did not consider the Facebook's actions as bias and those who consider it as biased (either ingroup-aiding bias [liberals] or ingroup-harming bias [conservatives]).

Turning to our second dependent variable, *Seriousness*, we see that those in the *no bias* group evaluated Facebook workers' actions as not very serious (*M*_*No Bias*_ = 2.61, *SD* = 1.30), those in the *ingroup-aiding bias* group evaluated these actions as more serious (*M*_*Ingroup-aiding Bias*_ = 3.80, *SD* = 1.47), while those in the *ingroup-harming bias* group had the highest seriousness evaluations (*M*_*Ingroup-harming Bias*_ = 5.17; *SD* = 1.66). The difference between the three groups is statistically significant (F(2, 155) = 32.66, *p* < .001), and employing the Scheffe *post-hoc* test we see that, congruent with our hypothesis, the difference in seriousness evaluations between the *ingroup-aiding bias* and *ingroup-harming bias* groups is statistically significant (*p* < .001). These results, graphically shown in Fig 4, are robust to various changes in model specifications (see Section E in S1 Appendix).

Overall, the findings of Study 2 provide further support for our theoretical expectations. Partisans who identify an ingroup-harming bias consider it as more serious and as more warranting a correction than partisans who identify an ingroup-aiding bias. Nonetheless, the possibility remains that results from our first two studies are due to self-selection, or, in the case of Study 2, to other confounders such as conservatives (liberals) generally being harsher (more lenient) in their evaluations, regardless of whether a political bias is ingroup-aiding or ingroup-harming. In the third study we addressed these concerns using a survey experiment, which randomly assigned respondents to either an ingroup-aiding bias or ingroup-harming bias condition.

### Study 3

#### Overview

This study was similar in its procedure and measures to Study 1, only this time respondents were randomly assigned to read either an article that is clearly in favor of Prime Minister Netanyahu, or against him. Importantly, in this study we used an "encouragement design" [54] in which random assignment was intended to encourage respondents to identify either an ingroup-harming bias or an ingroup-aiding bias, depending on the vote-choice of each respondent, thereby allowing us to test whether partisans react differently to the different types of bias. More specifically, randomly assigning our respondents to either a pro- or anti-Netanyahu article enables us to infer with greater confidence that varied responses to ingroup-harming bias and ingroup-aiding biases are not due to self-selection or any omitted variables, but rather due to motivated reasoning among partisans. The experiment was fielded between May 16 and 23, 2016.

#### Participants

A total of 344 Israeli respondents, recruited by the same survey company as in Study 1, participated in the study (Study 1 participants were restricted from participating). Sample characteristics are similar to those of Study 1: average age was 38.8 (*SD* = 12.8) and women constituted 50.3% of the sample. A plurality of respondents (41.6%) reported voting in the 2015 Israeli elections for right-wing parties, 22.4% for left-wing parties, 22.4% for center parties, and 13.7% either voted for other parties, did not vote, or refused to answer.

#### Procedure

Our intention was to make this study as comparable as possible to Study 1, while providing participants with either an article in favor of Netanyahu or an against him. We chose two articles that were published on October 2, 2015 following Netanyahu's speech at the United Nations General Assembly (see [55]). After slightly editing each article and shortening them to about 370–390 words, we successfully confirmed in a pilot study that the articles were indeed perceived as favorable or hostile towards Netanyahu as intended (see Section G in S1 Appendix). Participants were randomly assigned to read either of the two articles–presented as an article published during October 2015, one day after Netanyahu's speech at the United Nations, on one of Israel's top online news websites. As in Study 1, respondents then answered a 7-point "bias in the article" question and a follow-up question asking whether they would demand correction of the article.

#### Measurements

Our measures were almost identical to those used in Study 1. Pertaining to the dependent variable, *Demand for Correction*, 31.6% of partisans, i.e., voters of left- and right-wing parties, demanded a correction. The *Bias Type* measure, based on respondents’ answer to the 7-point "bias in the article" question and their vote in the elections, again had three categories: ingroup-aiding bias; no bias [neutral]; and ingroup-harming bias (see Study 1 above). Yet in this study we treated this measure as an ordinal-level variable (and not as a categorical variable): 0- ingroup-aiding bias; 1- no bias; 2- ingroup-harming bias. We did so in order to make the variable suitable for our statistical analyses (see below). Importantly, in this study, selection into these three categories was expected to be affected by the random allocation to either a pro- or anti-Netanyahu article. Overall, 37.1% of partisans identified an ingroup-aiding bias, 15.6% identified the article as neutral, and 47.3% identified an ingroup-harming bias. We also created a *Level of Bias* measure by folding the 7-point "bias in the article" scale at the midpoint (among partisans: *M* = 1.72; *SD* = 1.08).

#### Results and discussion

First, a series of randomization (balance) checks, detailed in Section G in S1 Appendix, confirm that our randomization was successful as our two groups (a pro- or anti-Netanyahu article) were adequately balanced. We also conducted manipulation checks. As intended, respondents assigned to the pro-Netanyahu article evaluated it as biased towards him (*M* = 3.43; *SD* = 1.84), deviating from the neutral score of 4 (*t*(175) = -4.14; *p* < .001), and respondents assigned to the anti-Netanyahu article perceived it as biased against him (*M* = 5.30; *SD* = 1.63), deviating from the neutral score of 4 (*t*(163) = 10.19; *p* < .001).

Next, we examined the Intent-to-Treat (ITT) estimate for each article by comparing the proportion of right- and left-wing voters who demanded a correction in each article (these results are also shown in a table format in Section I in S1 Appendix). As expected, in the pro-Netanyahu article the proportion of left-wing voters who demanded a correction, 30.6% [15.3%, 45.8%], was higher than that of right-wing voters, 13.2% [5.1%, 21.3%] (*χ*^2^(1) = 4.55; *p* = .033). And the difference between partisans was in the opposite direction in the anti-Netanyahu article: the proportion of left-wing voters who demanded a correction, 25.0% [10.7%, 39.3%], was now lower than that of right-wing voters, 54.6% [42.4%, 66.7%] (*χ*^2^(1) = 8.25; *p* = .004).

These ITT estimates, however, do not provide us with the quantity of interest with regard to our theoretical argument, namely, the difference in the proportion of demanding a correction between those who identified an ingroup-harming bias and an ingroup-aiding bias (or no bias). While our ordinal-level *Bias Type* variable is endogenous, in this study we can use random assignment to one of the two articles in order to instrument for *Bias Type*, thus enabling us to estimate the average treatment effect for those respondents who complied with the random assignment [56]. As our instrument, we created a *Hostile* dummy variable, based on the exogenous, randomly assigned article version and on partisan group affiliation (left-wing party or right-wing party). This variable takes the value 1 if the article is hostile toward the respective respondent’s political block (e.g., a left-wing voter receiving a pro-Netanyahu article), and 0 otherwise.

In addition to the exogeneity (or ignorability) of the instrument, another critical assumption underlying Instrumental Variable (IV) estimation is the exclusion restriction which requires that the instrument affect the outcome variable only through the instrumented endogenous variable [56]. A potential violation of this assumption in our study is an effect of the instrument through another post-treatment variable, namely, *Level of Bias*. Indeed, *Hostile* and *Level of Bias* are positively correlated (*Spearman rho* = .18; *p* = .008), and the *Level of Bias* and *Demand for Correction* variables are also positively correlated (*Spearman rho* = .27; *p* < .001). In Section H in S1 Appendix we detail several analyses we have conducted to assess–and consequently allay this threat to the exclusion restriction assumption.

Next, to test our hypothesis we fitted a two-stage least squares (2SLS) regression, controlling only for the two variables constituting the *Hostile* variable (a dummy variable denoting voting for left- or right-wing parties, and a dummy for the article version). From the first-stage regression (detailed in Section H in S1 Appendix) we see that *Hostile* strongly affects *Bias Type* (*t* = 8.11, *p* < .001), and that the model's F-statistic (*F*(1, 201) = 65.78, *p* < .001) satisfies the requirement that the instrument is sufficiently strong [56]. Results from the second-stage regression are presented in Table 2. Model 1 shows that, as expected, instrumenting for *Bias Type* indeed results in a significant effect on the *Demand for Correction* variable (*b* = .249; *p* < .001). These results provide strong support for our hypothesis.

**Table 2:** Study 3 –Second-stage estimates from the IV regression.

	(1)	(2)	(3)
Dependent Variable	Demand for Correction	Demand for Correction	Demand for Correction
Bias Type	.249***		
	(.069)		
Ingroup-harming Bias		.475***	.426**
		(.130)	(.146)
Moderate Bias			.165+
			(.084)
Strong Bias			.099
			(.088)
Left-right Vote (Right = 1)	-.011	-.003	.005
	(.063)	(.062)	(.061)
Article Version (Anti-Netanyahu = 1)	.211***	.214***	.216***
	(.062)	(.060)	(.059)
Constant	-.053	-.011	-.064
	(.086)	(.076)	(.066)
Observations	205	205	205
R^2^	0.154	0.184	0.217

*Note*. Robust standard errors in parentheses

*** p<0.001

** p<0.01

* p<0.05

+ p<0.1.

Nonetheless, Model 1 assumes that the effect of *Bias Type* is the same when moving from ‘ingroup-aiding bias’ to ‘no bias’ and when moving from ‘no bias’ to ‘ingroup-harming bias’. Accordingly, we created an alternative variable to be instrumented–an *Ingroup-harming Bias* dummy variable (1- ingroup-harming bias; 0- ingroup-aiding bias and no bias). Merging ‘no bias’ with ‘ingroup-aiding bias’ is justified based on the results of Study 1 in which no statistically significant difference was found between the ‘ingroup-aiding bias’ group and the ‘no bias’ group in the level of *Demand for Correction*. Similarly, in Study 3, 15.8% [7.5%, 24.0%] of the respondents who perceived ingroup-aiding bias demanded a correction, in comparison to 12.5% [0.9%, 24.1%] of those who perceived no bias (*χ*^2^(1) = 0.19, *p* = .66). Yet among those who perceived ingroup-harming bias, as much as 50.5% [40.5%, 60.5%] demanded correction. In Model 2 we thus use the *Ingroup-harming Bias* binary variable as our endogenous variable. Importantly, in the first-stage regression *Hostile* strongly affects *Ingroup-harming Bias* (*t* = 7.79, *p* < .001), with the model's F-statistic (*F*(1,201) = 60.76, *p* < .001) sufficiently strong (see Section H in S1 Appendix).

As expected, the results of Model 2 show that instrumenting *Ingroup-harming Bias* significantly increases *Demand for Correction* (*b* = .475; *p* < .001). Notably, the effect of this variable is almost double the effect of a one-unit increase in the three-category *Bias Type* variable depicted in Model 1. This finding, in line with Study 1's results, further suggests that in some cases it is the identification of an ingroup-harming bias, and not the identification of bias *per se*, that causes people to demand corrective action. Finally, to ascertain that the effect of the *Ingroup-harming Bias* variable is not a result of the level of bias a person perceived in the article, Model 3 adds controls for *Level of Bias* by adding the *Moderate Bias* and *Strong Bias* dummy variables (see Study 1 above). This addition hardly affects the substantive results, providing further support for our hypothesis.

In Section H in S1 Appendix we provide additional IV analyses in which we add several individual-level controls. As can be expected in an experimental study, these additions do not alter the main findings. Furthermore, we conducted a separate IV analysis for each article version to make sure that the baseline results are not due to only one of the article versions. The results of these analyses allay such concerns. Overall, the results of Study 3 provide experimental evidence in support of our hypothesis that partisans tend to react differently for ingroup-harming and ingroup-aiding biases. The following section assesses the causal mechanism underlying these findings.

### Assessing mechanisms: "Sincere" or "strategic" reactions to bias?

If partisans indeed evaluate and react differently to ingroup-harming and ingroup-aiding biases, two different explanations may be considered. The first assumes that partisans' reported evaluations of bias, as well as their reported reactions to it, reflect sincere evaluations and reactions. More specifically, that partisans first report their genuine perceptions of political neutrality or bias in a given act/message, and based on these perceptions, they report their genuine reaction to the neutral/biased message. However, another potential explanation is that partisans are in fact strategic in both their reported evaluations of bias and in their reactions to it. According to this explanation, partisans respond to both questions in a way that will improve the standing of their party, candidate or ideology, not necessarily reflecting authentic judgments and intentions. Indeed, there is some evidence suggesting that some claims by political elites of bias against their group are not entirely sincere but partly strategic ([57]; see also p. 139 in [58]).

Several studies provide evidence in support of the "sincere" account. Exposure to uncongenial news article was found to exert *actual* psychological discomfort, as manifested in increased levels of Cortisol, the "stress hormone" [59], and perceptions of bias are said to have actual detrimental effects, such as reduced trust in various institutions [16,17]. Moreover, as previous studies have shown (see, e.g., [42,53]), and as we document in Studies 2 and 3, when a certain act or message is clearly biased against or in favor of a certain party, a clear majority from *all* sides–including those who supposedly stand to benefit from that bias–agree as to the direction of that bias, a pattern that is inconsistent with a strictly strategic behavior.

**Fig 5:**
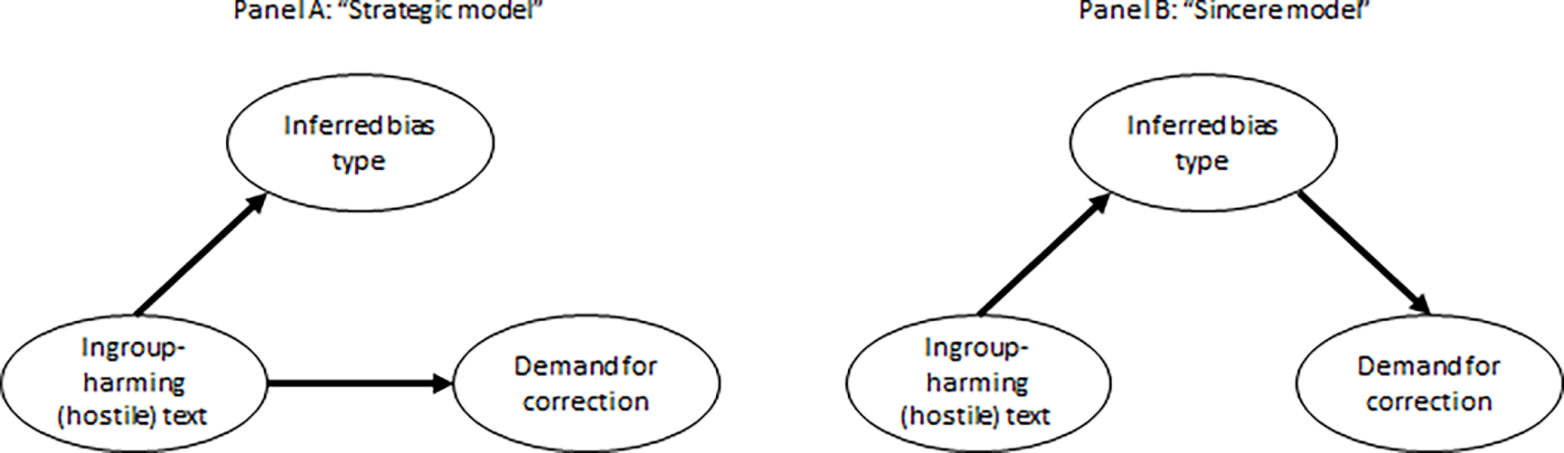
The "strategic" and "sincere" casual mechanisms.

A more direct assessment of the relative merit of the two explanations can be performed by contrasting the two distinct causal mechanisms they entail. The "strategic" model implies that after a partisan identifies the text as hostile toward her ingroup, she would tend to evaluated it as biased against her ingroup, and would evaluate this bias as more severe and warranting correction. Importantly, the hostility of the text is expected to affect *both* perceptions of bias and reactions to it. This causal mechanism is graphically presented in Panel A in Fig 5. The "sincere" model, on the other hand, implies that judgments of seriousness of the bias and the likelihood of demanding correction would be dominantly based on the perception of bias in the text, and not merely on the hostility of the text. More formally, the "sincere" model predicts that the effect of the text's hostility toward the respondent's ingroup on the likelihood of demanding correction and on the seriousness evaluations, would be mediated by her perception of (ingroup-harming) bias in the text, rather than affect these reactions directly. This model is presented in Panel B in Fig 5.

Based on the data of Study 3, Table 3 presents the results of a causal mediation analysis [60], which computes the average causal mediation effect (ACME), i.e., the expected change in the likelihood of demanding a correction when the mediator (bias-type) takes the value it would realize under the treatment condition (ingroup-hostile text), as opposed to the control condition (ingroup-supporting text), while the treatment status is held constant. The analysis was implemented via the mediation package in Stata [61]. To satisfy the sequential ignorability assumption [60], we include pretreatment controls (gender, age, education, religiosity, left/right political block voting, and the version of the article) that may be related to both the outcome (*Demand for Correction*) and the mediator (the ordinal, 3-category *Bias Type*), and we also control for *Level of Bias* as post-treatment confounder (the results are nearly identical if this variable is not included in the analysis).

**Table 3:** Causal mediation analysis.

	Treatment (*Hostile)*
Mediation effect [95% CI]	.112 [.041, .180]
Direct effect	.062 [-.072, .197]
Total effect	.174 [.052, .302]

*Note*. Mediation effects were calculated using an algorithm suggested by [61]. The outcome variable is *Demand for Correction*, the treatment is *Hostile*, and the mediator is *Bias Type*.

Table 3 demonstrates that exposure to an ingroup-hostile text increases the likelihood of demanding a correction by increasing the tendency to infer that the text is biased against one's ingroup. This mediated effect of *Bias Type* is positive and statistically significant, and this causal pathway is estimated to account for 65% of the total effect. On the other hand, the direct effect of exposure to the hostile text on the proclivity to demand a correction is smaller and statistically insignificant. These results are consistent with the "sincere" model, in the sense that merely being exposed to an ingroup-hostile text does not directly affect *Demand for Correction*, but only if such an exposure leads to an identification of an ingroup-harming bias.

It should be noted that the literature on perceptions of, and reactions to bias (either political, gender, racial or many other types of bias) has thus far overwhelmingly treated people's responses as genuine, and has not considered the possibility that some responses are strategic rather than sincere. Indeed, future research should provide more evidence as to the prevalence of strategic considerations in this literature.

## General discussion

In three studies we show that even though politically biased messages and acts are generally considered wrong and inappropriate, and while many people report that *prima facie* they prefer political neutrality over political bias in various social institutions even when a bias would help their ingroup, partisans react differently to ingroup-aiding and ingroup-harming biases; namely, they consider the latter as more serious and warranting a corrective action than the former. In line with the motivated reasoning literature (e.g., [11,12]) we show that partisans' normative evaluations and their likelihood of seeking corrective actions in response to a biased act or message, are influenced by their partisan affiliations. This is true both when partisans themselves evaluate bias in a certain news article (Studies 1 and 3) and when they evaluate allegations of bias leveled against other people or companies (Study 2).

Furthermore, in Studies 1 and 3 we see that partisans who identified an ingroup-aiding bias in a news item did not consider it more warranting a correction, in comparison to those partisans who identified no bias in that item (in Study 1 this is the case only after controlling for the level of bias). Given that respondents who perceived no bias provide the baseline estimates for demanding a correction of any action or message, these results not only suggest that partisans react differently to ingroup-aiding and ingroup-harming biases, but also that in some cases partisans do not appear to care much about politically biased acts or messages *per se*. Rather, they mostly care about political bias when the biased act or message is directed against their ingroup.

Our results have several potential implications. First, they demonstrate that even in the face of undisputed politically biased acts, political considerations and group loyalties seem to shape people's reactions to these act, thus reducing the likelihood of inter-party agreement on how to resolve such situations. We believe that the differences in partisans' reactions to ingroup-aiding and ingroup-harming political biases, demonstrated in this article, cast doubt on the possibility that rival partisans will collaborate in responding to what both sides see as blatant political bias against (or in favor of) a particular group. For example, should a newspaper's editorial board order a correction of a biased news item or not? How severe should be the treatment of, say, a public official whose decision or conduct was found to be politically biased (see, e.g., [62])? The answers to these and other related questions, it seems, are likely to be influenced by one's political preferences, even when the facts are not disputed.

Second, lack of inter-party agreement on how to address a given biased act could exacerbate inter-group tensions even more. Disparity between the corrective actions sought by the deprived group and those suggested by the group that gains from the undisputed biased act might dampen inter-group trust. Under such circumstances, the former group might be seen as trying to seize the moment and implement policies that would benefit it beyond what is supposedly necessary, and the latter group’s lenient reaction can give rise to claims of belittling a serious violation of the neutrality norm.

In order to mitigate these potential implications, we tentatively suggest that people from rival sides, perhaps in addition to non-partisans, should jointly decide how to deal with specific instances of overt bias. In the absence of such cooperation, each side might see the response to the bias as inapt–either too harsh or too lenient; and an intergroup cooperation could make both sides understand the feelings and perceptions of the opponent, which might inspire collectively agreed-upon action. Alternatively, partisans could be urged to "consider the opposite" [63] when deciding on the seriousness of the bias or the corrective act to be implemented; for example, how serious a partisan would consider an ingroup-aiding (ingroup-harming) bias had it been ingroup-harming (ingroup-aiding). Future research could determine whether these suggestions are indeed helpful and productive.

Third, this study contributes to the burgeoning literature regarding people's reactions to political bias. For example, Rojas has shown that partisans' perceptions of bias against the ingroup in news coverage are correlated with actions that "seek to enrich public debate and ‘correct’ what are seen as potential biases" (p. 1 in [18]). Our article contributes to this literature by showing that even prior to decisions regarding the proper response to perceived bias, rival partisans diverge on its seriousness, and that after encountering bias, the corrective actions people are likely to pursue depend on the target of that biased act. Noting the importance of people's reactions to bias (e.g., [25]), we believe that this article can set the stage for further research concerning reactions to political bias among people in general, and among partisans in particular. For example, in cases where the bias is so overt and overwhelming, partisans from rival groups might react similarly to it. Discovering such "boundary conditions" for the effect of partisans' motivations on their judgments could help us understand the nature of partisans' reactions to bias (see also [64]).

Finally, our findings may be extended to further types of inter-group conflicts. For example, would women and men react similarly to instances of bias against women in academic hiring (e.g., [65]), or would men consider such a bias as less serious and less warranting correction compared to women. The same goes for Caucasians and African-American in the U.S., as well as other ethnic groups in other countries. Such possibilities certainly await further research.

This article is not without limitations. First, all samples in this paper are online convenience samples. Thus, while several studies have recently shown that reliance on such samples could provide results similar to those of representative samples (e.g., [51,66,67]), we still believe that our results should also be replicated in nationally representative samples (but see [68]). Second, in Study 2 we do not have a clear-cut case of actual bias, but rather allegations of political bias. We have tried to circumvent this by asking respondents to consider the case under the assumption that the allegations were true, yet we cannot reject the possibility that in a case in which the biased act was known to be true, reactions would have been different. Third, our studies assessed partisans' reaction to bias only in the context of news and media coverage. Future studies could investigate partisans' reactions to bias in other contexts (academia, governmental agencies, international bodies, etc.). Finally, the designs of our three studies do not allow us to reject the possibility that some of our respondents' responses where more strategic than sincere. Future research could no doubt provide more insight into this possibility.

Despite these limitations, we believe that our consistent results across three contextually and methodologically diverse studies provide support for our theoretical expectation. Apart from the merit in replicating these results in other political spheres, we propose to research new interventions that might help mitigate partisans' biased reaction to political bias.

## Supporting information

S1 Appendix Supplementary materials. This document contains further information about the three studies as well as additional analyses.
(DOCX)

S1 Dataset Datasets and codes. This file contains all datasets and codes necessary to replicate the statistical analyses reported.
(ZIP)
