# Supplementary material for: When do we care about political neutrality? The hypocritical nature of reaction to political bias
Source: PLoS One. 2018 May 3;13(5):e0196674. doi: 10.1371/journal.pone.0196674 (PMC5933769; doi:10.1371/journal.pone.0196674)
Supplement: S1 Appendix — This document contains further information about the three studies as well as additional analyses. (DOCX) [file pone.0196674.s001.docx]

Supplemental Appendix for:

**When do we care about political neutrality?**

**The hypocritical nature of reaction to political bias**

--- For online publication only ---

**The appendix contains the following materials:**

Section A: Response Rates and Comparisons with Other Samples

Section B: The Texts of the Articles Used in the Three Studies

Section C: Robustness Tests for Study 1

Section D: Study 1 – Non-partisans

Section E: Robustness Tests for Study 2

Section F: Study 2 – Non-partisans

Section G: Study 3 – Pilot Study, Evaluations of Articles, and Balance Checks

Section H: Study 3 – Instrumental Variable (IV) Estimation

Section I: Study 3 – Non-partisans**Section A: Response Rates and Comparisons with Other Samples**

Section A.1 – The "participation rates" for the two Israeli samples:

The internet samples we used in Studies 1 and 3 are non-probability samples. Thus, we followed the American Association of Public Opinion Research's (AAPOR) recommendations regarding non-probability samples, and calculated the "participation rate" (pp. 49–50 in [1]), or "completion rate" (pp. 1021–1022 in [2]), for each of the two Israeli samples.

Study 1 was embedded in a larger sample which served additional research purposes. The survey company sent out a total of 9,959 invitations to answer the survey, and closed the survey after a pre-set quota had been filled. Overall, a total of 1,018 respondents provided "a usable response" (p. 49 in [1]); thus, the participation rate was 1,018 / 9,959 = 10.2%. Roughly one third of the survey's respondents (346) were part of Study 1. In Study 3, a total of 344 respondents provided a usable response. The survey company sent out a total of 3,067 invitations to answer the survey, and again closed the survey after a pre-set quota had been filled. The participation rate was thus 344 / 3,067 = 11.2%. We did not calculate participation rate for Study 2 (MTurk sample) since we do not know how many MTurk workers were exposed to our task's advertisement.

Section A.2 – Comparing the two Israeli samples with a representative sample:

In Table S1 we compare some of the demographic characteristics of our Israeli online samples (Studies 1 and 3) with those of a nationally representative sample conducted by the Israeli National Elections Study (INES) (<http://ines.tau.ac.il/2015.html>) between February and April 2015. As can be seen in Table S1, there is no meaningful difference between the characteristics of either of our two Israeli samples. Both samples, however, are somewhat dissimilar to the nationally representative sample. Participants in both our samples are younger and more supportive of the political right than participants in the nationally representative sample. Our samples also underrepresent Israel's non-Jewish, mostly Arab population. In contrast, our samples do resemble the nationally representative sample in terms of the proportion of women, the number of respondents who studied in college, and in terms of the levels of religious observance.

**Table S1. Comparing the Israeli samples with a nationally representative sample**

|  | Study 1's Sample (Jan. - Feb. 2016) | Study 3's Sample (May 2016) | INES 2015 National Sample |
| --- | --- | --- | --- |
|  |  |  |  |
|  |  |  |  |
| Age (Mean; *SD*) | 38.8 (*12.*6) | 38.8 (*12.*8) | 47.1 (*18.9*) |
| Women (% of sample) | 46.8% | 50.3% | 50.2% |
| Studied in College (% of sample) | 47.7% | 44.2% | 43.6% |
| Jewish (% of sample) | 93.5% | 97.4% | 80.7% |
| Observance of religious tradition |  |  |  |
| Not at all | 19.9% | 23.4% | 19.9% |
| A little bit | 40.5% | 40.6% | 43.9% |
| A lot | 25.9% | 22.2% | 24.7% |
| Observe all of it | 13.7% | 13.7% | 11.5% |
| Ideology (1-7 left-right ideological scale) |  |  |  |
| Right | 54.7% | 58.2% | 45.5% |
| Center | 24.3% | 26.6% | 24.8% |
| Left | 21% | 15.2% | 29.7% |
| Voted in the 2015 elections for |  |  |  |
| Right-wing parties | 41.3% | 41.6% | 38.0% |
| Center parties | 22.6% | 22.4% | 14.4% |
| Left-wing parties | 24.8% | 22.4% | 31.3% |
| Other / didn't vote | 11.3% | 13.7% | 16.3% |
|  |  |  |  |

*Note*. Data from the 2015 INES sample were extracted from the INES 2015 pre-election survey (February-March), except for the "voted in the 2015 election to" data, which was taken from the respective post-election survey (March-April).

Section A.3 – Comparing the MTurk sample with other MTurk samples:

In Table S2 we compare the demographic characteristics of our MTurk sample (Study 2) with those of other recent studies which relied on MTurk samples: [3–5]. While our sample's proportion of women is somewhat lower than those of two of the samples, we see that in the ideology and partisan identification parameters our sample is very similar to those of the other studies. In addition, our mean age is somewhat higher than the mean age of the other samples, and our sample also resembles both Clifford et al.'s and Levay et al.'s samples in term of education levels.

**Table S2. Comparing the MTurk Sample and Other MTurk Samples**

|  | Study 2's Sample (May 2016) | Berinsky et al.'s Sample (Feb. - March 2010) | Levay et al. 2016 (June 2013) | Clifford et al.'s Sample (June 2015) |
| --- | --- | --- | --- | --- |
|  |  |  |  |  |
|  |  |  |  |  |
| Age (Mean) | 37.3 | 32.3 | 31.6 | 34 |
| Women (% of sample) | 43.4% | 60.1% | 46.1% | 53% |
| Education |  |  |  |  |
| Less than high school | 0.5% | - | 1.3% | 2% |
| High school credential | 14.3% | - | 10.0% | 9% |
| Some college | 37.8% | - | 43.8% | 39% |
| Bachelor’s degree | 36.7% | - | 35.8% | 38% |
| Graduate degree | 10.7% | - | 9.1% | 12% |
| Ideology (1-7 Liberal-conservative scale) |  |  |  |  |
| Liberal | 54.9% | - | 58.4% | 59% |
| Moderate | 19% | - | 19.7% | 15% |
| Conservative | 26.2% | - | 21.9% | 25% |
| Ideology (Mean on a similar 7-point scale; 7- extreme conservative) | 3.35 | 3.39 | 3.30 | - |
| Party identification |  |  |  |  |
| Democrat | 45.9% | 40.8% | 46.1% | - |
| Independent | 33.7% | 34.1% | 34.3% | - |
| Republican | 18.9% | 16.9% | 14.6% | - |
| None / other | 1.5% | 8.2% | 4.9% | - |
|  |  |  |  |  |

*Note*. We could not compare our education estimates with those of Berinsky et al.'s sample since they reported mean years of education. Similarly, other blank rubrics are due to lack of data similar to our data in either sample.

**Section B: The Texts of the Articles Used in the Three Studies**

The original Hebrew articles from Studies 1 and 3 can be obtained upon request from the authors.

The full text of the article in Study 1:

**Wave of Terror: Netanyahu in Message to Citizens, Criticism Continues from the Left.**

**October 2015.**

Prime Minister Benjamin Netanyahu today convened a discussion and situation evaluation in the Jerusalem police operations room, with the participation of officials of the Ministry of Public Security, the Jerusalem Municipality, and the police. The discussion followed the cabinet decision yesterday to bolster Jerusalem forces, adding 1,000 policemen and border police, an increase, according to Netanyahu, intended to “calm the area”. Netanyahu admitted to police commanders, police officers and border police, “We are at the peak of a wave of terror. We are acting aggressively against the attacking terrorists, against lawbreakers and against inciters. We have augmented our forces and we are employing all means necessary to fight against this terror”.

The prime minister also appealed to the public: “I want to praise the restraint and courage shown by the citizens of Israel in recent days, as well as their behavior today. The aim of terror is to sow fear and our first mandate to defeat terror is to demonstrate calmness, and personal and national resilience. We have experienced more difficult times than these, and we will overcome this wave of terror with determination, responsibility and unity”.

Netanyahu’s response followed the second terror attack in several days, on Haggay Street in Jerusalem. In the Old City Muslim quarter, people are still fearful. “We greatly respect the police and we also admire them. But we demand that there be the right kind of security. There should be no possibility of an attack coming right after a previous one, as did the one which took place this morning. In the last few days, there has been stone throwing,” said Rachel Shapira, a resident of the Muslim quarter.

Parallel to Netanyahu’s statements and those of other government ministers, criticism is continuing from the political left at the stagnating political process. Opposition leader Yitzhak “Bougie” Herzog criticized the policies of the prime minister last night in a Knesset speech. Herzog related to Netanyahu’s and the government’s policies: “Netanyahu says that he is managing the conflict. The conflict you have been managing your way in the past few years has become a knife in the backs of the citizens of Israel. You have failed. The despicable terrorists have raised their heads during your guardianship. What you are proposing is a Masada, it’s living by the sword forever. I feel as though I am facing Prime Minister Golda Meir during the Yom Kippur War, after she had rejected every previous peace proposal,” added Herzog. “The writing is on the wall and you (Netanyahu) are like the three monkeys – hear no evil, see no evil, speak no evil. You, who have accused Livni and me of intending to divide Jerusalem, are, in fact dismantling it. Your policies of “managing the conflict” throughout your term of office have failed; they have crashed; they have exploded in all of our faces. Indeed, if I were prime minister, you would now be standing on the terrace in Zion Square and inciting giant demonstrations throughout the country.”

The full text of the article in Study 2:

Earlier this month, the website Gizmodo published a report suggesting that Facebook workers have deliberately and routinely suppressed news stories of interest to conservative users from the social network’s “trending” news section. According to a former journalist who worked on the project, workers prevented stories about various conservative topics – but not more liberal topics – from appearing in the highly-influential section, even though these conservative topics were originally trending among the site’s users. Following the accusations made by the former worker, the website Gizmodo has suggested that in fact, Facebook’s news section operates like a traditional newsroom, reflecting the biases of its workers, which is in stark contrast to the company’s claims that the trending module simply lists topics that have recently become popular on Facebook.

Officials from Facebook were quick to respond, denying any allegations of suppression of stories of interest to conservatives. The firm's co-founder and chief executive, Mark Zuckerberg, also denied allegations. He announced that Facebook will be conducting a full investigation to ensure the news section team upheld the integrity of that product, and stated that in order "to serve our diverse community" the firm is "committed to building a platform for all ideas".

The full text of the pro-Netanyahu article in Study 3:

**The World Loves Illusions, Netanyahu Describes Reality.**

**October 2015.**

And perhaps, just because of that, Netanyahu yesterday decided that “sometimes silence is the best response,” as the saying goes. If not for the dangers involved in the agreement, perhaps Netanyahu might even have been satisfied with a 44 second speech. But the thundering historical silence which echoed loudly yesterday at the UN General Assembly demanded words as well.

And considering those words, the head of the opposition, Bougie Herzog, was quick to conclude that Netanyahu had not made a speech of hope but rather a speech of despair. Less than an hour later, a murderous terror attack took place in Samaria, when an Israeli couple was murdered as their four young children sat terrified in the back seat of the car. Anyone living in this country actually knows the reality in which we live. Netanyahu’s role at the General Assembly was to describe this situation, even if the world does not like to listen – and not to sell the illusions that the world so loves to hear.

Netanyahu, who could have torn the lie-laden speech of the Palestinian Authority president to shreds, chose yesterday to present Israel’s aspirations for peace to the world. In these troubled days, the prime minister chose to integrate glimmers of hope. But while the prime minister was speaking, the Samaria terrorists had already sought easy Jewish prey, perhaps in order to celebrate the Palestinian flag-raising which took place two days ago at the United Nations.

And that’s exactly what Netanyahu was trying to explain. We are living in a Middle East which is becoming more and more dangerous and the security challenges Israel must deal with are only becoming greater. The State of Israel cannot remain indifferent to these threats, and certainly not to the Iranian threat, and it is fed up with the indifference of the rest of the world. That was the message. Has it penetrated? That is not certain. Was it transmitted? In the best and clearest way.

The prime minister understands precisely which way the winds are blowing, and now, after the agreement with Iran has been signed, he is expecting that the world will track the Iranians, pay attention to their activity against Israel and he expects the world to battle Iranian terror. Netanyahu well knows the power of the American president. He is perhaps disappointed with Obama (and made sure to clarify that in his criticism of the agreement) but he hopes to see Obama become a control tower for the implementation of the agreement with Iran. At least that. We would have preferred to hear a different speech from the prime minister. We would have preferred that a different agreement had been signed with Iran. We would have preferred that the world be a bit less hypocritical. We would have preferred that there had not been another terror attack last night. “People don’t believe lies because they have to, but because they want to,” observed British journalist and author Malcolm Muggeridge, and that’s exactly what the world is doing as they face Iran, as they face Abu Mazen.

And that is precisely the reason Netanyahu remained silent for 44 seconds.

The full text of the anti-Netanyahu article in Study 3:

**Netanyahu’s Speech: Somewhere between Pathetic and Annoying.**

**October 2015.**

For his admirers, Prime Minister Benjamin Netanyahu’s speech yesterday at the UN General Assembly delivered the goods. In an “I accuse” without apology, Netanyahu tore the curtain away from a hypocritical world, condemned the nuclear agreement with Iran, held out a hand to Mahmoud Abbas, despite his lies, and ended with the brilliant gimmick of a thundering silence from the podium which certainly would grab the headlines and go viral. From their standpoint, it was Bibi at his best.

But on the other hand, for Netanyahu’s critics, this speech arouses anger and incredulity: for its bombast and pompousness, and the shticks and tricks that Netanyahu cannot do without; his taunting of his host, the United Nations, his arrogant tone, and his vitriolic tongue-lashing of the world powers who had signed the agreement with Iran, and the stale demand for negotiations “with no prior conditions” as if the world was created yesterday, as if it was not completely clear that in this way, the cycle of bloodshed which yesterday claimed even more victims, would continue indefinitely.

The speech caused despair among senior Democrats and the American government, which had hoped that Netanyahu would moderate a bit his castigation of the Iran agreement after he had failed to block it, and would recognize the need to concentrate efforts against the Islamic state. The speech certainly did not supply even a bit of hope for those who always wish for some new proposal or dramatic program which will at last justify the claim put forth for thirty years that “Netanyahu will yet surprise us.” The speech also did not relieve American Jews who now suffer from post-trauma at the rift which has opened among them in the struggle against the nuclear agreement, who pray for its end, closing the circle, and returning the demons to their bottle.

It was a speech which convinced the convinced and deepened the doubts of the doubters from the outset, but it also had a slightly pathetic element. Netanyahu was indeed speaking to a half-empty hall of a Generally Assembly which had actually ended, while outside, the police had already removed the barricades and inside, the corridors were emptying. As Netanyahu rose to speak, a few bored journalists from Gabon and Mongolia were sitting in the main hall and they barely raised their eyes to what was going on in front of them. In that sense, Netanyahu was caught in a situation he could not have wanted, somewhere between pathetic and annoying. He was pathetic as he had fascinated the world up until the confirmation of the nuclear agreement with Iran and now he was forced to supply entertainment from the sidelines. He was annoying because he was perceived as someone whose aim in life was to bore humanity.

Because, while the universe understands that the deal with Iran has already entered the history books, Netanyahu feels that that it is “the greatest moral imperative” to prove for the who-knows-how-many-times, that justice lies with him. Because, while the world fears a potential apocalyptic conflict between the titans, Russia and the United States, in Syria, Netanyahu bargains and settles accounts with his super-pathetic neighbor, Abu Mazen, about prayer arrangements on the Temple Mount. Because, in the absence of a desire for peace or hope for change, the two of them, Netanyahu and Abu Mazen, sound like two bothersome individuals whose audiences are just waiting for the gong which will signify that their time has passed.**Section C: Robustness Tests for Study 1**

In Table S3 we present several models aimed at testing the robustness of the basic regression model we present in Model 2 of Table 1 in the main text. First, in Model 1 in Table S3 we present the full results of Model 3 of Table 1 in the main text. In this model we add several individual-level variables, including age, gender, religion (Jewish / non-Jewish), college education, and political interest. As noted in the main text, adding these variables hardly affects the results. Second, in our baseline models we considered partisan respondents who answered "don't know" to our *Demand for Correction* question as people who did not demand a correction. In Model 2 in Table S3 we omit these respondents (N = 38) from the regression analysis in order to make sure results are not sensitive to the omission of the Don't Know responses, and to the inclusion of only those who demanded a correction and those who approved the article as is. The results are even stronger in this model specification: the coefficient of the *Ingroup-harming Bias* category (*b* = 3.06) is slightly bigger than its respective coefficient in Model 2 in Table 1 in the main text (*b* = 2.22).

**Table S3. Robustness Tests for Table 1 in the Main Text**

|  | (1) | (2) | (3) | (4) | (5) | (6) |
| --- | --- | --- | --- | --- | --- | --- |
|  | w. Individual-level controls | w/o DK responses | w/o too-fast answers I | w/o too-fast answers II | Interaction w. IMC | Interaction w.  questionnaire version |
|  |  |  |  |  |  |  |
| Ingroup-aiding Bias | 0.926 | 1.355+ | 0.938 | 0.876 | -0.490 | 0.895 |
|  | (0.749) | (0.701) | (0.699) | (0.755) | (1.498) | (0.872) |
| Ingroup-harming Bias | 2.217*** | 3.057*** | 2.209*** | 2.350*** | 2.276** | 2.007** |
|  | (0.576) | (0.620) | (0.556) | (0.588) | (0.877) | (0.734) |
| Moderate Bias | 0.804 | 0.136 | 0.633 | 0.774 | 0.780 | 0.747 |
|  | (0.526) | (0.568) | (0.507) | (0.509) | (0.504) | (0.495) |
| Strong Bias | 1.162+ | 0.256 | 0.965+ | 1.165* | 1.084* | 0.986+ |
|  | (0.600) | (0.595) | (0.526) | (0.550) | (0.552) | (0.517) |
| Age | -0.018 |  |  |  |  |  |
|  | (0.016) |  |  |  |  |  |
| Gender (female=1) | -0.174 |  |  |  |  |  |
|  | (0.391) |  |  |  |  |  |
| Jewish | -1.119 |  |  |  |  |  |
|  | (1.263) |  |  |  |  |  |
| Studied in College | 0.761+ |  |  |  |  |  |
|  | (0.407) |  |  |  |  |  |
| Political Interest | 0.308 |  |  |  |  |  |
|  | (0.245) |  |  |  |  |  |
| Religiosity | 0.157 |  |  |  |  |  |
|  | (0.198) |  |  |  |  |  |
| Ideological Strength | -0.183 |  |  |  |  |  |
|  | (0.212) |  |  |  |  |  |
| IMC (Passed=1) |  |  |  |  | 0.095 |  |
|  |  |  |  |  | (0.895) |  |
| Ingroup-aiding Bias X IMC |  |  |  |  | 1.808 |  |
|  |  |  |  |  | (1.554) |  |
| Ingroup-harming Bias X IMC |  |  |  |  | -0.185 |  |
|  |  |  |  |  | (1.022) |  |
| Version (1= w/o bias) |  |  |  |  |  | -0.194 |
|  |  |  |  |  |  | (0.845) |
| Ingroup-aiding Bias X Version |  |  |  |  |  | -0.325 |
|  |  |  |  |  |  | (1.249) |
| Ingroup-harming Bias X Version |  |  |  |  |  | 0.397 |
|  |  |  |  |  |  | (0.968) |
| Coefficient equality F-tests (p-value) |  |  |  |  |  |  |
| H0: Ingroup-aiding Bias = Ingroup-harming Bias | .026 | .001 | .012 | .006 | - | - |
| Constant | -2.466 | -2.578*** | -2.663*** | -2.901*** | -2.803*** | -2.639*** |
|  | (1.749) | (0.425) | (0.423) | (0.460) | (0.730) | (0.599) |
|  |  |  |  |  |  |  |
| Observations | 204 | 170 | 194 | 199 | 208 | 208 |
| Pseudo R2 | 0.260 | 0.279 | 0.215 | 0.249 | 0.232 | 0.219 |

*Note*. Robust standard errors in parentheses; *** p<0.001, ** p<0.01, * p<0.05, + p<0.1.

Third, in Model 3 we excluded respondents who potentially answered the survey too quickly. As the entire survey contained almost 1100 words, we opted to consider respondents who answered faster than two minutes as respondents answering the survey too quickly. Removing these respondents hardly affects the results. Fourth, in Model 4 we excluded participants whose time of answering the last question on the webpage in which the news article appeared was either too fast or too slow (i.e., two standard deviations below the mean time or above it). Again, results hardly change.

Fifth, in one of the questionnaire questions we used an Instructional Manipulation Check (IMC) [6], intended to detect satisficing and carelessness in answering the survey questions. Overall, 135 partisans (64.9%) passed the IMC. Since omitting 35.1% of partisans (73 respondents) could reduce statistical power, in order to determine the sensitivity of our basic model to passing the IMC we chose to separately interact our two dummy variables, *Ingroup-aiding Bias* and *Ingroup –harming Bias*, with an IMC dummy variable. Results are presented in Model 5, and we can see that these two interactions are not statistically significant.

Finally, in Study 1 we used two versions of our bias question. Respondents were randomly assigned to receive either a version of the questionnaire in which the 7-point bias question included the word "biased" (as shown in the main text), or another version that used a similar question, employed earlier by several scholars (e.g., [7,8]), in which the word *bias*ed was omitted, i.e., the article is either *in favor of* or *against* *Netanyahu*.

A series of randomization checks we performed (not shown) indicate that regressing this binary questionnaire version variable on a series of individual-level variables (those included in Model 1 in Table S3, in addition to a variable capturing the 2015 election vote) fails to product significant results (the model's chi-squared test *χ*²(10) = 9.9; *p* = .45). Partisans did not differ in the *Level of Bias* variable between the two questionnaire versions (*M_version_bias_* = 1.11, *SD* = 1.12; *M _version_favor_* = 0.85; *SD* = 1.11) (*t*(203.36) = 1.63; *p* = .105). Yet they did differ in the 7-point "bias in the article" variable (*M_version_bias_* = 4.69, *SD* = 1.41; *M _version_favor_* = 4.23; *SD* = 1.39) (*t*(204.22) = 1.63; *p* = .017), suggesting that using the word "bias" (or "biased") caused partisans to report slightly more biased coverage against Prime Minister Netanyahu. Further research, we suggest, might help elucidate the potential effect of linguistic differences on people's report of bias.

In addition, while among partisans in the "bias" questionnaire version, 40.2% identified a neutral article, in the other, "favor" questionnaire version, the corresponding figure is 56.3%. Partisans in the "bias" questionnaire version also identified more ingroup-aiding bias (20.5%) and ingroup-harming bias (39.3%) in the article than partisans in the "favor" questionnaire version (11.5% and 32.3%, respectively) (*χ*²(2) = 6.11; *p* = .047). Nonetheless, there is no significant difference between the two questionnaire versions in terms of our dependent variable, *Demand for Correction*, where in the "bias" questionnaire version 25.9% partisans demanded a correction in comparison to 21.9% in the other questionnaire version (*χ*²(1) = 0.46; *p* = .50).

Still, our main concern is that these differences will affect partisans' demand for a correction of the article. In order to examine this, we separately interacted the *Ingroup-aiding* *Bias* and *Ingroup-harming* *Bias* dummy variables with a dummy variable for questionnaire version. The results, presented in Model 6, show that these interaction terms are statistically insignificant, assuaging concerns that our results are driven by the different question-wording. All in all, our robustness tests provide further support for the baseline estimates presented in the main text.

**Section D: Study 1 - Non-partisans**

We now turn to examine the effect of reporting bias on *non-partisans*. Overall, among non-partisans who identified a neutral article, 8.7% demanded a correction, and among those who identified any bias, that proportion rose to 23.0% (*χ*²(1) = 3.81; *p* = .051). In Table S4 we run two binary logistic regressions in order to determine whether the relationship between identifying any bias (*Bias*: 0- no bias; 1- any level of bias) holds even when controlling for *Level of Bias* (namely, the *Moderate Bias* and *Strong* *Bias* dummy variables). In Model 2 we see that adding these dummy variables renders the *Bias* coefficient statistically insignificant; that is, it is mostly seeing higher levels of bias that renders non-partisans likely to demand a correction, rather than simply identifying a bias.

**Table S4. Study 1 – Non-partisans and Demand for Correction**

|  | (1) | (2) |
| --- | --- | --- |
| Dependent Variable | Demand for Correction | Demand for Correction |
|  |  |  |
| Bias | 1.140+ | 0.594 |
|  | (0.608) | (0.716) |
| Moderate Bias |  | 0.659 |
|  |  | (1.258) |
| Strong Bias |  | 1.129+ |
|  |  | (0.656) |
| Constant | -2.351*** | -2.351*** |
|  | (0.526) | (0.526) |
|  |  |  |
| Observations | 107 | 107 |
| Pseudo R2 | 0.0418 | 0.0739 |

*Note*. Robust standard errors in parentheses; *** p<0.001, ** p<0.01, * p<0.05, + p<0.1.

**Section E: Robustness Tests for Study 2**

Section E.1 – Robustness tests of the main results:

In Table S5 we present several models in which we check the robustness of Study 2's baseline results. In Models 1–3 we perform analyses regarding the *Change Algorithm* variable, whereas in Models 4–5 we perform analyses regarding the *Seriousness* variable. We start with the analyses regarding the *Change Algorithm* variable. First, in Model 1 we add several individual-level variables, namely, age, gender, ideological strength, and education, to our baseline model. Adding these variables in Model 1 hardly changes the statistical significant of the *Ingroup-aiding Bias* or the *Ingroup-harming Bias* dummy variables. The difference between these two dummy variables is still statistical significant (*p* = .019). Second, in Model 2 we exclude respondents who potentially answered the survey too quickly. The entire survey contained slightly less than 700 words, and so we opted to consider respondents who answered quicker than one minute as respondents answering the survey too quickly. Removing these five respondents hardly affects the results, with the difference between these two dummy variables still statistical significant (*p* = .035).

**Table S5. Robustness Tests for Study 2's Main Results**

|  | | (1) | (2) | (3) | (4) | (5) |
| --- | --- | --- | --- | --- | --- | --- |
| Dependent variable | Change Algorithm - w. Individual-level controls | | Change Algorithm - w/o too-fast answers | Change Algorithm - w/o DK responses | Seriousness - w. Individual-level controls | Seriousness - w/o too-fast answers |
|  |  | |  |  |  |  |
| Ingroup-aiding Bias | 2.444*** | | 2.253*** | 2.527*** | 1.170*** | 1.309*** |
|  | (0.493) | | (0.476) | (0.562) | (0.265) | (0.271) |
| Ingroup-harming Bias | 3.741*** | | 3.510*** | 4.397*** | 2.623*** | 2.711*** |
|  | (0.670) | | (0.650) | (1.097) | (0.317) | (0.318) |
| Age | -0.018 | |  |  | -0.018 |  |
|  | (0.024) | |  |  | (0.013) |  |
| Gender | 0.348 | |  |  | -0.316 |  |
|  | (0.430) | |  |  | (0.236) |  |
| Ideological Strength | -0.385 | |  |  | -0.128 |  |
|  | (0.236) | |  |  | (0.193) |  |
| Education | 0.043 | |  |  | 0.028 |  |
|  | (0.169) | |  |  | (0.097) |  |
| Constant | -0.204 | | -1.135** | -0.636 | 3.536*** | 2.459*** |
|  | (1.086) | | (0.384) | (0.414) | (0.662) | (0.206) |
| Coefficient equality F-tests (p-value) |  | |  |  |  |  |
| Ingroup-aiding Bias =  Ingroup-harming Bias | 0.019 | | 0.035 | 0.085 | 0.000 | 0.000 |
| Observations | 158 | | 153 | 131 | 158 | 153 |
| R-squared |  | |  |  | 0.321 | 0.319 |
| Pseudo R2 | 0.278 | | 0.242 | 0.307 |  |  |
| Model's Significance (p-value) | 0 | | 0 | 0 |  |  |
| Adj. R-Squared |  | |  |  | 0.294 | 0.310 |

*Note.* Robust standard errors in parentheses. *** p<0.001, ** p<0.01, * p<0.05, + p<0.1

In Model 3 we run the basic model only this time we omit from the analysis those respondents (N = 27) who answered "Don't know" in the *Change Algorithm* question (i.e., leaving only those who either supported or did not support such a change). The coefficients of the *Ingroup-aiding Bias* or the *Ingroup-harming Bias* dummy variables are even larger in this model, albeit the standard errors in this model are larger, slightly reducing the statistical significance of the difference between these two dummy variables (*p* = .085).

In Models 4–5 we run similar analyses, only this time we examine the sensitivity of the *Seriousness* variable. In Model 4 we add the individual-level variables mentioned above. Adding these variables hardly affects the results, with conservatives who identified ingroup-harming bias still seeing the Facebook's actions as significantly more serious than liberals who identified ingroup-aiding bias. Finally, in Model 5 we exclude respondents who potentially answered the survey too quickly. Again, removing these respondents hardly affects the results. In both these models the differences between the *Ingroup-aiding Bias* and the *Ingroup-harming Bias* dummy variables are statistically significant (*p*s < .001).

Section E.2 – Results among republicans and democrats:

While in the main text we compared the results of conservatives and liberals, in this section we show that results are rather similar – albeit slightly weaker – if we compare the results of republican and democrat respondents.

We start with the first dependent variable, *Change Algorithm*. Among republicans and democrats who did not consider the alleged actions of Facebook workers as political bias against conservatives, i.e., the *no bias* group, only 30.7% [16.1%, 45.4%] thought that Facebook should change its algorithm if the allegations are found true. In comparison, in the *ingroup-aiding bias* group (i.e., democrats who considered Facebook workers' actions as bias against conservatives) 78.1% [69.0%, 87.1%] thought a change to Facebook's algorithm is in place, and in the *ingroup-harming bias* group (republicans who considered Facebook workers' actions as bias against conservatives) that figure was 90.9% [82.3%, 99.5%]. The difference between the three groups is statistically significant (*χ*²(2) = 40.50; *p* < .001), and, importantly, the difference between the *ingroup-aiding* and *ingroup-harming* groups is also marginally significant (*χ*²(1) = 3.29; *p* = .070).

Turning to our second dependent variable, *Seriousness*, we see that republican and democrat respondents in the *no bias* group evaluated Facebook workers' actions as not very serious (*M_No Bias_* = 2.54, *SD* = 1.27), those in the *ingroup-aiding bias* group evaluated these actions as more serious (*M_Ingroup-aiding Bias_* = 3.87, *SD* = 1.50), while those in the *ingroup-harming bias* group had the highest seriousness evaluations (*M_Ingroup-harming Bias_* = 5.07; *SD* = 1.65). The difference between the three groups is statistically significant (F(2, 162) = 29.77, *p* < .001), and employing the Scheffe *post-hoc* test we see that, congruent with our hypothesis, the difference in seriousness evaluations between the *ingroup-aiding* *bias* and *ingroup-harming bias* groups is statistically significant (*p* < .001).

**Section F: Study 2 – Non-partisans**

In the main text of Study 2, we only analyzed partisans' (i.e., liberals and conservatives) reactions to the allegations made against Facebook workers, namely, their evaluation of the seriousness of these actions and whether Facebook should change its algorithm. We now elaborate about non-partisans' (i.e., ideological moderates) responses, and compare them to those of partisans.

First, 75.7% of ideological moderates considered the actions of Facebook workers as political bias against conservatives (if the allegations were found to be true). That estimate is somewhere in the middle between conservatives and liberals, as among conservatives the respective estimate is 92.2% while among liberals the respective estimate is 65.4%. The difference between the three groups is statistically significant (*χ*²(2) = 12.99; *p* = .002).

Pertaining to the first dependent variable, *Change Algorithm*, we see that 85.7% of ideological moderates who considered the actions as bias against conservatives answered that Facebook should change its algorithm if the allegations were true, in comparison to 75.7% of liberals and 91.5% of conservatives. Again, that estimate is somewhere in the middle between the two other ideological group, albeit slightly closer to conservatives. And pertaining to the second dependent variable, *Seriousness*, we see that overall, ideological moderates who considered the actions as bias against conservatives evaluated these actions as somewhat serious (*M* = 4.82; *SD* = 1.66). Again, that estimate is somewhere in the middle between liberals and conservatives who considered the actions as bias against conservatives (*M_Liberals_* = 3.80, *SD* = 1.47; *M_Conservatives_* = 5.17; *SD* = 1.66), even though it is closer to conservatives' evaluations.

**Section G: Study 3 – Pilot Study, Evaluations of Articles, and Balance Checks**

Section G.1 – Pilot study for Study 3:

In our Study 3's pilot study we asked respondents to evaluate the bias in the pro-Netanyahu and anti-Netanyahu articles. Thirteen respondents evaluated each article. Respondents in both groups were, on average, slightly more left-wing than right-wing, with no statistically different ideological scores for each group. Those in the pro-Netanyahu article had an average ideology score of 4.92 on a 1-7 left-right ideological scale (7- extreme left) (*SD* = 1.3), and those in the anti-Netanyahu article had an average ideology score of 5.31 (*SD* = 1.55) (*t*(22.9) = 0.682; *p* = .25).

The 13 respondents who evaluated the pro-Netanyahu article found it biased in favor of Netanyahu (*M* = 2.31; *SD* = 1.44), while a one-sample t-test confirmed that this result was significantly different from the "neutral" score of 4 (*t*(12) = -4.25; *p* < .001). The 13 respondents who evaluated the pro-Netanyahu article found it biased against Netanyahu (*M* = 5.77; *SD* = 0.44), as a one-sample t-test confirmed that this result was significantly different from the "neutral" score (*t*(12) = 14.53; *p* < .001).

Importantly, both right-wing and left-wing respondents in this pilot study identified the pro-Netanyahu article as biased in his favor (bias < 4) and the anti-Netanyahu article as biased against him (bias > 4). In the pro-Netanyahu article we had 3 right-wing respondents (ideology < 4) who identified the article as biased in favor of Netanyahu (bias < 4), and in the anti-Netanyahu article we had 10 left-wing respondents (ideology > 4) who identified the article as biased against Netanyahu (bias > 4). Thus, we can conclude that our articles were indeed very much one-sided so that our results are not highly affected by partisans' tendency to see media coverage as biased against their side.

Section G.2 – Partisans' evaluations of the pro- and anti-Netanyahu articles:

In this section we elaborate about partisans' evaluations of the bias in the pro- and anti-Netanyahu articles in Study 3. First, the pro-Netanyahu article was indeed seen as favorable towards him (*M* = 3.43; *SD* = 1.84), deviating from the neutral score of 4 (*t*(175) = -4.14; *p* < .001). However, left-wing voters considered the article as more biased in favor of Netanyahu (*M* = 2.75; *SD* = 1.84) than right-wing voters (*M* = 3.62; *SD* = 1.79) (*t*(72.97) = 2.35; *p* = .022). In addition, the anti-Netanyahu article was indeed seen as biased against him (*M* = 5.30; *SD* = 1.63), deviating from the neutral score of 4 (*t*(163) = 10.19; *p* < .001). Interestingly, left-wing voters (*M* = 5.34; *SD* = 1.37) and right-wing voters (*M* = 5.44; *SD* = 1.70) identified the article as similarly biased against Netanyahu (*t*(85.5) = -0.31; *p* = .76). This latter finding, which could be the result of rather harsh language used in the article – for example, in describing Netanyahu and his speech the author used, *inter* *alia*, Hebrew words which translate into "pathetic" and "annoying" – provides us with an instance in which partisans from rival sides agreed on both the direction *and* the level of bias.

Section G.3 – Balance checks for Study 3:

To make sure that random assignment to the two articles was successful, we need to make sure that the respondents in the two articles versions do not significantly differ in any of the individual-levels variables. Therefore, we examined whether these variables differed in their means between the two articles: we conducted chi-square tests for the dichotomous and ordinal demographic variables (*Gender*, *Studied in College*, and vote in the 2015 Israeli election or ideological group [left-wing, center, right-wing]) and independent t-tests for the other variables (*Age* and *Religiosity*). Results (not shown) indicate that indeed, respondents in the two articles were adequately balanced with regard to these individual-levels variables as none of the tests conducted was statistically significant.

In addition, we fitted a Logistic regression in which the dependent variable was the article version (pro-/ anti-Netanyahu) in order to investigate whether the individual-levels variables predict the chances of being in one of the article versions (see Table S6). As can be seen in Model 1, in which we add dummy variables for vote for center parties and vote for left-wing parties in the 2015 Israeli Election variable (vote for right-wing parties as reference category), the model as a whole is statistically insignificant (*p* = .91). In Model 2 we replace the vote-choice variables with dummy variables for affiliation for either the ideological center or the ideological left (ideological right as reference category). Again, the model is statistically insignificant (*p* = .96). These results confirm that our randomization was successful.

**Table S6. Balance Checks for Study 3**

|  | (1) | (2) |
| --- | --- | --- |
| Dependent Variable | Version | Version |
|  |  |  |
| Age | 0.010 | 0.010 |
|  | (0.009) | (0.009) |
| Gender (female=1) | -0.053 | -0.046 |
|  | (0.227) | (0.225) |
| Studied in College | -0.064 | -0.048 |
|  | (0.229) | (0.229) |
| Religiosity | 0.074 | 0.051 |
|  | (0.138) | (0.126) |
| Vote for Center | -0.249 |  |
|  | (0.330) |  |
| Vote for Left | 0.090 |  |
|  | (0.344) |  |
| Other Vote / Didn't Vote | -0.050 |  |
|  | (0.361) |  |
| Ideological Center |  | -0.067 |
|  |  | (0.271) |
| Ideological Left |  | -0.091 |
|  |  | (0.357) |
| Constant | -0.564 | -0.512 |
|  | (0.572) | (0.519) |
|  |  |  |
| Observations | 320 | 324 |
| Pseudo R^2^ | 0.00624 | 0.00338 |
| Models' Chi-Squared Statistic | 2.757 | 1.512 |
| Chi-Squared Statistic's (p-value) | 0.907 | 0.959 |

*Note*. Robust standard errors in parentheses. In Model 1 voting for right-wing parties is the reference category, and in Model 2 it is the Ideological Right category.

**Section H: Study 3 – Instrumental Variable (IV) Estimation**

Section H.1 – Potential risks to the Exclusion Restriction:

An important assumption underlying IV estimation is the exclusion restriction which requires that the instrument affects the outcome variable only through the instrumented endogenous variable [9]. As noted in the main text, a potential threat to this assumption in our study is an effect of the instrument through another post-treatment variable, namely, *Level of Bias*, as both *Hostile* and *Level of Bias* variables are positively correlated, as are *Level of Bias* and *Demand* *for Correction*.

In order to address this threat to the exclusion restriction assumption, and following the rationale provided by Gerber and colleagues (pp. 737–742 in [10]), we ran a mediation analysis to directly test this causal path. Causal mediation analysis [11] provides an estimate of the average causal mediation effect (ACME), i.e., the expected change in our dependent variable (*Demand for Correction*) when the potential mediator (*Level of Bias*) takes the value it would realize under the treatment condition (*Hostile*=1) as opposed to the control condition (*Hostile*=0), while the treatment status is held constant. The analysis was implemented via the Stata mediation package [12]. To satisfy the sequential ignorability assumption [11], we included the other endogenous variable (*Bias Type*) as well as the two dummy variables constituting the *Hostile* variable (a dummy variable denoting voting for either left- or right-wing parties, and a dummy for the article version), these are variables that may be related to both the outcome (*Demand for Correction*) and the mediator (*Level of Bias*).

The mediated effect of the *Level of Bias* variable is positive, but statistically insignificant (.024; 95% CIs: -.005, .064). Similar results were obtained using the *Ingroup-harming Bias* dummy instead of the *Bias Type* variable (see the main text); the mediated effect of the *Level of Bias* variable in this specification is negative and statistically insignificant (-.002; 95% CIs: -.026, .020). These results do not support the proposition that the treatment effect was mediated by *Level of Bias* – and consequently allay this concern regarding the exclusion restriction assumption.

Importantly, we also conducted the opposite analyses, namely, separately testing whether the *Bias Type* and the *Ingroup-harming Bias* variables mediate the effect of the *Hostile* variable on the *Demand for Correction* dependent variable, while controlling for the *Level of Bias* variable and the other two control variables. As expected, in both analyses the mediated effect is positive and statistically significant. The mediated effect of the *Bias Type* variable is .119 [95% CIs: .050, .191], and the mediated effect of the *Ingroup-harming Bias* variable is .104 [95% CIs: .045, .168].

Section H.2 – Estimates from the IV regressions:

Table S7 below presents the results of the first-stage estimates of the Instrumental Variable (IV) regressions detailed in Table 2 in the main text. In addition, as a robustness test we reran these IV regressions once more, only this time we omitted the respondents who answered "Don't know" to the *Demand for Correction* questions (N = 31). The results of these analyses (not shown) are very similar to the original results in the main text.

**Table S7. First-stage Estimates from the IV Regressions in the Main Text**

|  | (1) | (2) | (3) |
| --- | --- | --- | --- |
| Dependent Variable | Bias Type | Ingroup-harming Bias | Ingroup-harming Bias |
|  |  |  |  |
| Hostile | .928*** | .485*** | .437*** |
|  | (.114) | (.062) | (.064) |
| Moderate Bias |  |  | .197* |
|  |  |  | (.081) |
| Strong Bias |  |  | .260*** |
|  |  |  | (.070) |
| Left-Right vote | .278* | .128* | .129* |
|  | (.114) | (.062) | (.061) |
| Article Version | -.116 | -.067 | -.070 |
|  | (.114) | (.062) | (.061) |
| Constant | .516*** | .181** | .076 |
|  | (.114) | (.062) | (.069) |
|  |  |  |  |
| Observations | 205 | 205 | 205 |
| R^2^ | 0.262 | 0.234 | 0.288 |
| First-stage F-statistic | 65.78 | 60.76 | 46.48 |
| F-statistic (p-value) | .000 | .000 | .000 |

*Note*. Robust standard errors in parentheses; *** p<0.001, ** p<0.01, * p<0.05, + p<0.1.

In addition, as explained in the main text, we also run additional IV analyses in which we added to our baseline models (Models 1–2 in Table 2 in the main text) several individual-level controls, namely, age, gender, education level (college education), and religiosity. Results, presented in Table S8, suggest that adding these variables does not affect the baseline results.

**Table S8: Estimates from the IV Regressions – With Individual-level Controls**

|  | (1) | (2) | (3) | (4) |
| --- | --- | --- | --- | --- |
| Dependent Variable | First-stage:  Bias Type | Second-stage:  Demand for Correction | First-stage:  Ingroup-harming Bias | Second-stage:  Demand for Correction |
|  |  |  |  |  |
| Hostile | .922*** |  | .487*** |  |
|  | (.114) |  | (.063) |  |
| Bias Type |  | .250*** |  |  |
|  |  | (.070) |  |  |
| Ingroup-harming Bias |  |  |  | .473*** |
|  |  |  |  | (.130) |
| Left-Right vote | .287+ | -.012 | .143+ | -.008 |
|  | (.149) | (.084) | (.080) | (.083) |
| Article Version | -.111 | .216*** | -.059 | .216*** |
|  | (.115) | (.061) | (.063) | (.061) |
| Age | -.004 | -.001 | -.004 | -.001 |
|  | (.004) | (.003) | (.002) | (.003) |
| Gender (female=1) | -.003 | .064 | .030 | .048 |
|  | (.109) | (.061) | (.062) | (.060) |
| Studied in College | -.273* | .060 | -.068 | .024 |
|  | (.113) | (.066) | (.062) | (.061) |
| Religiosity | -.050 | .005 | -.031 | .007 |
|  | (.064) | (.039) | (.035) | (.039) |
| Constant | .899*** | -.073 | .395** | -.036 |
|  | (.237) | (.167) | (.136) | (.161) |
|  |  |  |  |  |
| Observations | 205 | 205 | 205 | 205 |
| R^2^ | 0.287 | 0.162 | 0.249 | 0.188 |
| First-stage F-statistic | 64.91 |  | 60.18 |  |
| F-statistic (p-value) | .000 |  | .000 |  |

*Note*. Robust standard errors in parentheses; *** p<0.001, ** p<0.01, * p<0.05, + p<0.1.

Furthermore, we also run a separate analysis for each article version in order to ascertain that our baseline IV results are not due to an effect of only one of the article version (either the Pro- or Anti-Netanyahu article version) (the dummy variable denoting voting for either left- or right-wing parties was omitted due to collinearity). We run separate analyses for the *Bias Type* variable as well as for the *Ingroup-harming Bias* variable. The results are shown in Table S9 below, with Models 1–4 showing the results for *Bias Type* and Models 5–8 showing the results for *Ingroup-harming Bias*.

**Table S9: Estimates from the IV Regressions – For the Two Article Versions**

| Article Version | Pro-Netanyahu | | Anti-Netanyahu | | Pro-Netanyahu | | Anti-Netanyahu | |
| --- | --- | --- | --- | --- | --- | --- | --- | --- |
|  | (1) | (2) | (3) | (4) | (5) | (6) | (7) | (8) |
| Dependent Variable | First-stage: Bias Type | Second-stage: Demand for Correction | First-stage: Bias Type | Second-stage: Demand for Correction | First-stage: Ingroup-harming Bias | Second-stage: Demand for Correction | First-stage: Ingroup-harming Bias | Second-stage: Demand for Correction |
|  |  |  |  |  |  |  |  |  |
| Hostile | .650*** |  | 1.206** |  | .358*** |  | .613*** |  |
|  | (.177) |  | (.145) |  | (.097) |  | (.078) |  |
| Bias Type |  | .266* |  | .239** |  |  |  |  |
|  |  | (.125) |  | (.083) |  |  |  |  |
| Ingroup-harming Bias |  |  |  |  |  | .484* |  | .470** |
|  |  |  |  |  |  | (.223) |  | (.159) |
| Constant | .794*** | -.079 | .400*** | .162 | .309*** | -.017 | .114* | .203* |
|  | (.108) | (.127) | (.117) | (.109) | (.057) | (.097) | (.054) | (.093) |
|  |  |  |  |  |  |  |  |  |
| Observations | 104 | 104 | 101 | 101 | 104 | 104 | 101 | 101 |
| R^2^ | 0.113 | 0.096 | 0.409 | 0.073 | 0.118 | 0.130 | 0.341 | 0.109 |
| First-stage F-statistic | 13.51 |  | 68.95 |  | 13.49 |  | 62.45 |  |
| F-statistic (p-value) | .000 |  | .000 |  | .000 |  | .000 |  |

*Note*. Robust standard errors in parentheses; *** p<0.001, ** p<0.01, * p<0.05, + p<0.1.

Overall, these results suggest that there are no meaningful differences in the effect of the two endogenous variables on the *Demand for Correction* dependent variable across the two article versions. In both analyses, the effect of the exogenous *Hostile* variable on either endogenous variable (i.e., the first-stage estimates) is stronger in the Anti-Netanyahu article than in the Pro-Netanyahu article (compare Model 1 with Model 3, and Model 5 with Model 7). Still, the first-stage F-statistic of each of these models, despite the relatively low-powered analysis, is sufficiently strong (i.e., > 10; [9]). Moreover, the second-stage estimates of the two endogenous variables are statistically significant and, more importantly, are also very similar across the two article versions (compare Model 2 with Model 4, and Model 6 with Model 8). This suggests that the effect of the two endogenous variables is not dependent on an effect of a certain article version, and, more generally, that this effect is not dependent on the political group (i.e., voters of right- or left-wing parties) which suffers from an ingroup-harming bias or gains from an ingroup-aiding bias.

**Section I: Study 3 – Non-partisans**

In the main text of Study 3, we only analyzed partisans' (i.e., Israelis who either voted for the political left or the political right) responses, mainly their demand for a correction of the pro- and anti-Netanyahu articles. We now elaborate about non-partisans' responses and their demands for a corrective action, and compare them to those of partisans. Similar to Study 1, we considered non-partisans as those respondents who reported voting in the 2015 Israeli election to parties which do not tap into the right or left political blocs in Israel, as well as respondents who reported that they did not vote in that election. To facilitate the interpretation of the results, we present the results across the two article versions in Table S10.

**Table S10: Demands for a correction among partisans and non-partisans**

|  | Left-wing partisans | Non-partisans | Right-wing partisans | **Total** |
| --- | --- | --- | --- | --- |
| *Readers of the pro-Netanyahu article* | 30.6%  [15.3%; 45.8%]  (36) | 20.3%  [10.3%; 30.2%]  (64) | 13.2%  [5.1%; 21.3%]  (68) | 19.6%  [13.6%; 25.7%]  (168) |
| *Readers of the anti-Netanyahu article* | 25.0%  [10.7%; 39.3%]  (36) | 40.4%%  [26.9%; 53.9%]  (52) | 54.6%  [42.4%; 66.7%]  (66) | 42.9%  [35.0%; 50.7%]  (154) |
| ***Total*** | 27.8%  [17.4%; 38.2%]  (72) | 29.3%  [21.0%; 37.6%]  (116) | 33.6%  [25.5%; 41.6%]  (134) | 30.7%  [25.7%; 35.8]  (322) |

*Note*. Cell entries represent the percentage of respondents from each group who, after reading one of the articles, demanded a correction of the article. In brackets we report the 95% confidence intervals of these estimates, and in parenthesis we report the number of respondents in each cell. Left-wing partisans who were assigned to read a pro-Netanyahu article as well as right-wing partisans who were assigned to read an anti-Netanyahu are considered to have been encouraged to receive an ingroup-*harming* bias. Similarly, left-wing partisans who were assigned to read an anti-Netanyahu article as well as right-wing partisans who were assigned to read a pro-Netanyahu are considered to have been encouraged to receive an ingroup-*aiding* bias.

As noted in the main text, in the *pro*-Netanyahu article we saw that among partisans, respondents who voted for left-wing parties demanded more correction (30.6%) than those voting for parties of the right (13.2%). Non-partisans, we note, are somewhere in the middle, with 20.3% of non-partisans demanding a correction. While the difference between the two partisan groups was statistically significant, the difference between the three groups (left, non-partisans, and right) only approaches statistical significance (*χ*²(2) = 4.50; *p* = .105). Yet the addition of this third group renders this analysis underpowered. In the *anti*-Netanyahu article, we saw that respondents who voted for the left were now demanding correction of the article at lower rates than those who voted for the right (54.6% and 25.0%, respectively). Again, non-partisans were somewhere in the middle, between the two partisan groups, with 40.4% of non-partisans demanding a correction. In this article, the difference between the three groups (left, non-partisans, and right) is statistically significant (*χ*²(2) = 8.50; *p* = .014).

All in all, these results show that, as can be expected, non-partisans' demands for correcting a slanted article were both less severe/restrictive than partisans who suffered from that article and less lenient than partisans who gained from that article.

**References**

1. American Association of Public Opinion Research (AAPOR). Standard definitions: Final Dispositions of case codes and outcome rates for surveys. 9th edition. AAPOR. 2016.

2. Callegaro M, Disogra C. Computing response metrics for online panels. Public Opin Q. 2008;72: 1008–1032. doi:10.1093/poq/nfn065

3. Berinsky AJ, Huber GA, Lenz GS. Evaluating online labor markets for experimental research: Amazon.com’s Mechanical Turk. Polit Anal. 2012;20: 351–368. doi:10.1093/pan/mpr057

4. Clifford S, Jewell RM, Waggoner PD. Are samples drawn from Mechanical Turk valid for research on political ideology? Res Polit. 2015;2: 1–9. doi:10.1177/2053168015622072

5. Levay KE, Freese J, Druckman JN. The demographic and political composition of Mechanical Turk samples. SAGE Open. 2016;6: 1–17. doi:10.1177/2158244016636433

6. Oppenheimer DM, Meyvis T, Davidenko N. Instructional manipulation checks: Detecting satisficing to increase statistical power. J Exp Soc Psychol. Elsevier Inc.; 2009;45: 867–872. doi:10.1016/j.jesp.2009.03.009

7. Gunther AC, Christen CT. Projection or persuasive press? Contrary effects of personal opinion and perceived news coverage on estimates of public opinion. J Commun. 2002;52: 177–195. doi:10.1093/joc/52.1.177

8. Choi J, Yang M, Chang JJ. Elaboration of the Hostile media phenomenon: The roles of involvement, media skepticism, congruency of perceived media influence, and perceived opinion climate. Communic Res. 2009;36: 54–75. doi:10.1177/0093650208326462

9. Sovey AJ, Green DP. Instrumental variables estimation in political science: A readers’ guide. Am J Pol Sci. 2011;55: 188–200. doi:10.1111/j.1540-5907.2010.00477.x

10. Gerber AS, Huber GA, Washington E. Party Affiliation, partisanship, and political beliefs: A field experiment. Am Polit Sci Rev. 2010;104: 720–744. doi:10.1017/S0003055410000407

11. Imai K, Keele L, Tingley D, Yamamoto T. Unpacking the Black box of causality: Learning about causal mechanisms from experimental and observational studies. Am Polit Sci Rev. 2011;105: 765–789. doi:10.1017/S0003055411000414

12. Hicks R, Tingley D. Causal mediation analysis. Stata J. 2011;11: 1–15.
